# A new *Heraclides* swallowtail (Lepidoptera, Papilionidae) from North America is recognized by the pattern on its neck

**DOI:** 10.3897/zookeys.468.8565

**Published:** 2014-12-23

**Authors:** Kojiro Shiraiwa, Qian Cong, Nick V. Grishin

**Affiliations:** 1 12416 Picrus Street, San Diego, CA, USA 92129; 2 Howard Hughes Medical Institute; 3 Departments of Biophysics and Biochemistry, University of Texas Southwestern Medical Center, 5323 Harry Hines Blvd, Dallas, TX, USA 75390-9050

**Keywords:** Biodiversity, cryptic species, DNA barcodes, Neotropical, *Heraclides homothoas*, *Heraclides melonius*, butterfly release, APHIS

## Abstract

*Heraclides rumiko* Shiraiwa & Grishin, **sp. n.** is described from southwestern United States, Mexico, and Central America (type locality: USA, Texas, Duval County). It is closely allied to *Heraclides cresphontes* (Cramer, 1777) and the two species are sympatric in central Texas. The new species is diagnosed by male genitalia and exhibits a nearly 3% difference from *Heraclides cresphontes* in the COI DNA barcode sequence of mitochondrial DNA. The two *Heraclides* species can usually be told apart by the shape and size of yellow spots on the neck, by the wing shape, and the details of wing patterns. “Western Giant Swallowtail” is proposed as the English name for *Heraclides rumiko*. To stabilize nomenclature, **neotype** for *Papilio cresphontes* Cramer, 1777, an eastern United States species, is designated from Brooklyn, New York, USA; and lectotype for *Papilio thoas* Linnaeus, 1771 is designated from Suriname. We sequenced DNA barcodes and ID tags of nearly 400 Papilionini specimens completing coverage of all *Heraclides* species. Comparative analyses of DNA barcodes, genitalia, and facies suggest that *Heraclides oviedo* (Gundlach, 1866), **reinstated status**, is a species-level taxon rather than a subspecies of *Heraclides thoas* (Linnaeus, 1771); and *Heraclides pallas* (G. Gray, [1853]), **reinstated status**, with its subspecies *Heraclides Papilio bajaensis* (J. Brown & Faulkner, 1992), **comb. n.**, and *Heraclides anchicayaensis* Constantino, Le Crom & Salazar, 2002, **stat. n.**, are not conspecific with *Heraclides astyalus* (Godart, 1819).

## Introduction

Swallowtails (Papilionidae Latreille, [1802]) are arguably the best-known and best-studied butterflies due to their large size, dazzling colors, and elegant shapes. Despite significant research efforts, the family is still plagued with taxonomic difficulties and is rich in evolutionary puzzles (Zakharov et al. 2004, Kawahara and Breinholt 2014). From the origins of mimicry to speciation through hybridization, swallowtails are becoming a model group for evolutionary biology and genomics (Kunte et al. 2011, Zhang et al. 2013, Kunte et al. 2014). Groundbreaking work of Rothschild and Jordan (1906) established the foundation for the classification and future studies of Neotropical Swallowtails. Pioneering molecular studies by Sperling’s group shed light on their phylogeny and speciation, and revealed unsuspected complexities in relationships within the family (Sperling and Harrison 1994, Caterino and Sperling 1999, Zakharov et al. 2004, Simonsen et al. 2011). Tyler, Brown and Wilson (1994) summarized the knowledge on American Papilionidae in an amazingly instructive and compact volume. Nevertheless, the family is continuing to surprise us with its complexity and nuances (Lewis et al. 2014).

A multifaceted pattern of speciation resulted in 7 or 8 species of tiger swallowtails (Pterourus glaucus group) in North America (Kunte et al. 2011, Warren et al. 2014). The most remarkable of recent discoveries was the description of *Pterourus appalachiensis* Pavulaan & D. Wright, 2002, which is a hybrid species recently originated through gene exchange between *Pterourus canadensis* (Rothschild & Jordan, 1906) and *Pterourus glaucus* (Linnaeus, 1758) (Pavulaan and Wright 2002, Kunte et al. 2011, Zhang et al. 2013). *Pterourus canadensis* was originally proposed as a subspecies of *Pterourus glaucus*, but is treated today as a biologically distinct species differentiated from *Pterourus glaucus* about 600,000 years ago (Zhang et al. 2013). While the two species can hybridize over the narrow zone where they meet, they show significant divergence in a number of characters, both in genotype and phenotype (Hagen et al. 1991, Ording 2008, Ording et al. 2009, Winter and Porter 2010, Kunte et al. 2011, Zhang et al. 2013).

The Giant Swallowtails (Heraclides cresphontes group sensu Fig. 15, see below for more detailed definition) has received less attention. Currently treated as three species: *Heraclides cresphontes* (Cramer, 1777) distributed from Canada to Panama; *Heraclides homothoas* (Rothschild & Jordan, 1906) recorded from Costa Rica to Venezuela and Trinidad; and *Heraclides melonius* (Rothschild & Jordan, 1906), a Jamaican endemic, these butterflies are closely related to *Heraclides thoas* (Linnaeus, 1771) with its seven described subspecies ranging from Texas and Cuba to Uruguay and Argentina, and *Heraclides paeon* (Boisduval, 1836), consisting of three named subspecies known from Yucatan to Chile and Bolivia (Tyler et al. 1994, Lamas 2004, Warren et al. 2014). In agreement with Lewis et al. (2014), we find that the Greater Antilles endemics *Heraclides aristor* (Godart, 1819) and *Heraclides caiguanabus* (Poey, [1852]) are closely allied to the above-mentioned species, despite their striking difference in appearance due to the lack of a central band on their wings.

In 2005, KS collected several specimens of *Heraclides cresphontes* on lantana flowers near an orange orchard grove in Pauma Valley, San Diego County, California. Comparison with *Heraclides cresphontes* specimens from Silver Lake, Kosciusko County, Indiana revealed wing pattern differences between California and Indiana populations. Several years ago, with the accumulation of COI DNA barcode sequences in databases, NVG noticed that the only available sequence of *Heraclides cresphontes* from northeastern USA (Caterino and Sperling 1999) differed by about 3% from *Heraclides cresphontes* sequences from Costa Rica (Ratnasingham and Hebert 2007). While provoking, it was unclear how DNA sequences vary with large geographic distances. These anecdotal observations prompted further investigation. Dissection of male genitalia of *Heraclides cresphontes* from across its range revealed two groups consistent with pattern differences. Eastern *Heraclides cresphontes* differed from southwestern ones. DNA barcode sequences of 200 *Heraclides cresphontes* specimens from Canada to Panama and from California to Florida also fell in two sequence groups: the eastern and the southwestern haplotypes. There was very little variation within each haplotype group, but a 3% difference between the groups. Both haplotypes were present in central Texas. These results on correlation between genitalia, patterns and DNA barcodes suggested that the butterfly known as *Heraclides cresphontes* is a complex of two cryptic species, one of which was unnamed and is described herein.

## Materials and methods

Specimens used in this study were collected in the field under the permit #08-02Rev from Texas Parks and Wildlife Department to NVG, or examined in the following collections: Texas A&M University Collection, College Station, TX, USA (TAMU); University of Texas at Austin Insect Collection, Austin, TX, USA (TMMC); National Museum of Natural History, Smithsonian Institution, Washington, DC, USA (USNM); Colorado State University Collection, Fort Collins, CO, USA (CSUC); The Field Museum of Natural History, Chicago, IL, USA (FMNH); American Museum of Natural History, New York, NY, USA (AMNH); McGuire Center for Lepidoptera and Biodiversity; Gainesville, FL, USA (MGCL); San Diego Natural History Museum, San Diego, CA, USA (SDMC); and Los Angeles County Natural History Museum, Los Angeles, CA, USA (LACM) and several private collections. Standard entomological techniques were used for dissection (Robbins 1991), i.e., the abdomen or its distal part was broken off, soaked for 40 minutes (or until cleared) in 10% KOH at 60°C, dissected, and subsequently stored in a small glycerol-filled vial on the pin under the specimen. Genitalia and wing venation terminology follows Miller (1987) and Lewis (2010). Length measurements are in metric units and were made from photographs of specimens taken with a scale and magnified on a computer screen. Photographs of immature stages and specimens were taken with a Nikon D800 camera through a 105 mm f/2.8G AF-S VR Micro-Nikkor lens; dissected genitalia were photographed in glycerol with a Nikon D80 through 105 mm f/2.8G AF-S VR Micro-Nikkor lens and Nikon D200 camera without a lens and through microscopes at 1.5×–3× magnification. Images were assembled and edited in Photoshop CS5.1. Genitalic photographs were taken in several focus slices and stacked in Photoshop and Zerene Stacker to increase depth of field.

Legs (cut with scissors into tiny pieces in lysis buffer), crumbs and pieces of muscle tissue from the thorax of dissected specimens (plucked from the abdomen attachment site), or a distal part of an abdomen (dropped into lysis buffer, and after overnight incubation at 56°C transferred into 10% KOH for genitalia dissection) were used to extract genomic DNA with Macherey-Nagel (MN) NucleoSpin® tissue kit following the manufacturers protocol. The lysis buffer volume was scaled down to 70 μl for legs and volumes of subsequent reagents were proportionally reduced. Genomic DNA was eluted in a total volume of 40-100 μl MN BE buffer (concentration of DNA as measured by Promega QuantiFluor® dsDNA System was from near 0 to 20 ng/μl, mostly around 1 ng/μl, depending on specimen age and storage conditions) and was stored at -20°C.

PCR was performed using Invitrogen AmpliTaq Gold 360 master mix in a 20 μl total volume containing less than 1 ng of temfig DNA (usually 0.5–1 μl of DNA extract) and 0.5 μM of each primer. For legs from freshly collected specimens or those preserved in alcohol, the following primers were used to obtain the complete barcode: LepF: 5’-TGTAAAACGACGGCCAGTATTCAACCAATCATAAAGATATTGG-3’ and LepR: 5’-CAGGAAACAGCTATGACCTAAACTTCTGGATGTCCAAAAAATCA-3’. For older specimens (up to 1960) the following pairs of primers were used: swtl-COIF (forward, 5’-TTATTCAACAAATCATAAAGATATCGGA-3’) – swtl-mCOIR (reverse, 5’-GTTCCKGCYCCATTTTCTAC-3’), or sCOIF (forward, 5’-ATTCAACCAATCATAAAGATATTGG-3’) – smCOIR (reverse, 5’-CCTGTTCCAGCTCCATTTTC-3’), and swtl-mCOIF (forward, 5’-GACTTTTACCCCCTTCTCTAACTC-3’) – swtl-COIR (reverse, 5’-AAAATATAAACTTCAGGATGTCCAAA-3’), to amplify the barcode in two overlapping segments.

The barcodes of even older specimens (1900–1960) were amplified in four overlapping segments with the following four pairs of *Heraclides*-specific primers: paeon-COIF (forward, 5’-TCAACAAATCATAAAGATATCGGAAC-3’) – swtl-bCOIR (reverse, 5’-AATCAATTTCCAAATCCTCCAA-3’), swtl-bCOIF (forward, 5’-CCGGCTCATTAATTGGAGATG-3’) – swtl-mCOIR (reverse, 5’-CTGTTCCKCTYCCATTTTCTAC-3’), swtl-mCOIF2 (forward, 5’-TTTTGACTTTTACCCCCTTCTCTAA-3’) – swtl-eCOIR (reverse, 5’-CCTACGGCTCAAACAAATAAAGG-3’), and swtl-eCOIF (forward, 5’-TTCCTCAATTCTTGGRGCAATTA-3’) – swtl-COIR2 (reverse, 5’- AAAATATAAACTTCAGGATGTCCAAAAA -3’). In case of failure, additional primers that match target sequences better were used, as specified in GenBank entries for these sequences (KP173713–KP174107) and barcodes were amplified in more than 4 segments.

For some old specimens (e.g., 1870–1960), amplification of longer DNA segments failed. To obtain their sequences for identification, we developed *Heraclides*-specific primers for very short, about 100 bp fragments, which we call ID tags. A region in which the two USA *Heraclides* species differ from each other the most, was selected and the following primers were designed: swtl-ID1F (forward, 5’-TGAGCAAGAATACTAGGAACTTCTCTTA-3’) – swtl-ID1R (reverse, 5’-AATAAAAGCATGAGCTGTAACAATAGTA-3’) to amplify 64 bp sequence from the specimen (together with both primers, the actual product is 120 bp).

The PCR reaction was cleaned up by enzymatic digestion for the whole barcode amplifications, ID tag amplification, and sequences amplified in more than 2 segments, with 4 μl Shrimp Alkaline Phosphatase (20 U/μl) and 1 ul Exonuclease I (1 U/μl) from New England Biolabs. For sequences obtained in two segments, due to the frequent presence of primer dimers and other short non-specific PCR products, Agencourt Ampure XP beads or Invitrogen E-Gel® EX Agarose Gels (followed by Zymo gel DNA recovery kit) were used to select the DNA products of expected length. Sequences were obtained using the M13 primers (for amplification from LepF and LepR primers): 5’-TGTAAAACGACGGCCAGT-3’ or 5’-CAGGAAACAGCTATGACC-3’ or with primers used in PCR. Sanger sequencing was performed with Applied Biosystems Big Dye Terminator 3.1 kit on ABI capillary instrument in the DNA Sequencing Core Facility of the McDermott Center at UT Southwestern. The resulting sequence traces were proofread in FinchTV <http://www.geospiza.com/Products/finchtv.shtml>.

As a result, we obtained complete or partial DNA barcode sequences from 395 Papilionini specimens. Sequences and accompanying specimen data were submitted to GenBank and received accession numbers KP173713–KP174107. Data about these specimens are provided in Suppl. material 1.

Additional DNA sequences for analysis were downloaded from GenBank <http://genbank.gov/> (Benson et al. 2014) using accession numbers provided in Lewis et al. (2014) or were found by BLAST <http://blast.ncbi.nlm.nih.gov/> searches (Altschul et al. 1990) using sequences obtained by us to query “nr/nt” database, or from the BOLD database <http://boldsystems.org> (Ratnasingham and Hebert 2007). All sequences were aligned manually since they matched throughout their length without insertions or deletions. For a quick reference, Phylogeny.fr server at <http://www.phylogeny.fr/> was used with the Hamming distance model (Dereeper et al. 2008) to compute evolutionary distances from aligned DNA sequences and BioNJ (Gascuel 1997) algorithm to build dendrograms. A more thorough analysis was performed on DNA alignments using an elaborate comparative protocol involving a battery of methods and locally installed programs. In the analysis, because there are no insertions or deletions in the barcodes of butterflies, gaps (e.g., missing or ambiguous base pairs, including terminal ones) were treated as missing characters. TNT (version 1.1, available with the sponsorship of the Willi Hennig Society) was used for Maximum parsimony reconstruction (Goloboff et al. 2008). “New technology search” algorithm was used with Sect. Search, Ratchet (10 iterations), Drift (10 cycles), and Tree Fusing options enabled. Both Bremer support (Bremer 1994) and Bootstrap values were computed and mapped on the trees with TNT. Maximum Likelihood analysis was performed using RAxML (version 7.0.4) under several substitution models, such as GTRCAT, GTRGAMMA, and GTRGAMMAI (Stamatakis 2006). Rapid RAxML bootstrap values (-x option, and “-f a” for complete analysis) were computed to judge about the confidence of tree nodes. Bayesian Inference was performed with MrBayes v3.2.1 (Huelsenbeck and Ronquist 2001, Ronquist and Huelsenbeck 2003). Models with 1, 2 and 6 states were used (nst=1, 2, 6), with optimized fraction of invariant positions (propinv), gamma distribution parameter (gamma) or both (invgamma). The COI alignment was treated as a single partition, or analyzed as 3 partitions by codon position. Generations were carried out until convergence (standard deviation of split frequencies less than 0.01) and the first 25% were discarded as “burn in”. Posterior probabilities of nodes computed by MrBayes were used as the indicators of confidence.

## Results

Comparison of *Heraclides cresphontes* male genitalia throughout its range from Canada to Panama reveals two groups (Fig. 14). Specimens from the eastern group (Fig. 11B–D) possess shorter and more robust uncus arms. Brachium arms project from the base of uncus on the outer side and are mostly hidden under uncus in dorsal view. In lateral view, uncus and brachium point in the same direction. The bases of uncus and brachium are fused, brachium is stronger sclerotized at the base. Specimens from the southwestern group (Fig. 11b–d) are characterized by longer and more slender uncus arms, often strongly curving inwards. Brachium arms project from the base of uncus on the inner side, and are visible below uncus in dorsal view. In lateral view, uncus and brachium point away from each other: posterodorsad (uncus) and posteroventrad (brachium). The bases of uncus and brachium are weakly fused, with weaker sclerotization at the base of brachium.

Specimens from the eastern North America, from Canada to Florida and central Texas, USA belong to the eastern group. Specimens from other parts of the range from central Texas to California, USA and southwards to Panama belong to the southwestern group. In central Texas, both groups are present. Genitalic differences between groups correlate with the differences in facies (Fig. 11E, facies vs. genitalic differences on the plots). While facies are variable and some exceptions are found, the eastern group specimens have pale-yellow spots on the head, patagia, and thorax(Fig. 11A), but southwestern specimens are characterized by continuous yellow stripes instead (Fig. 11a). Southwestern specimens have generally narrower, less scalloped wings, longer and more slender hindwing tails, and exhibit statistical difference in details of wing patterns, mostly on ventral hindwing.

Correlation between genitalic and facies differences and geographic distribution suggests that we are dealing with two distinct evolutionary lineages diversified sufficiently to be treated as two taxonomic units. However, these units are mostly allopatric and overlap over narrow range in central Texas. Moreover, in the range of overlap, we see specimens with intermediate characters. Therefore, it was not clear whether to regard these taxonomic units as subspecies, or suggest that the divergence between them is sufficient for biological species.

To address this question, we determined COI mitochondrial DNA barcode sequences for 249 *Heraclides cresphontes*-like specimens from over 100 localities covering the entire distribution range from Canada to Panama. The results were surprisingly clear-cut. Each specimen fell into one of the two groups: eastern and southwestern (Figs 17, 18). Within each group, variability in barcode sequences was very low, within 0.5%, despite the range covering 3,000 miles, and was rather individual than geographic. E.g., specimens from San Diego, California (USA) had the same DNA barcode as those from the Cape region in Baja California Sur (Mexico), Yucatan (Mexico), or near Panama Canal (Panama). Between the two groups, there was a hiatus of nearly 3%.

To put this number in perspective and compare it with divergence in other Papilionini Latreille, [1802], DNA barcode differences between *Heraclides androgeus* (Cramer, 1775) and *Heraclides thersites* (Fabricius, 1775), *Pterourus glaucus* (Linnaeus, 1758) and *Pterourus canadensis* (Rothschild & Jordan, 1906), and *Papilio polyxenes* Fabricius, 1775 and *Papilio zelicaon* Lucas, 1852 are 2.9%, 2.1%, and 3.4% respectively. Distances between these species from three Papilionini genera are of the same magnitude as the distance between eastern and southwestern *Heraclides cresphontes* populations. On the other hand, barcode differences between Papilionini taxa regarded as subspecies are usually within 1.5%, and mostly below 1%. Three percent difference in the barcode suggests (April et al. 2013, Papadopoulou et al. 2009, Zhang et al. 2013) that the two *Heraclides cresphontes*-like taxa diverged from each other from one to three million years ago, prior to divergence between *Papilio glaucus* and *Papilio canadensis* (about 600,000). Thus, it is likely that eastern and southwestern *Heraclides cresphontes* populations have evolved in isolation long enough to fully speciate. These two species are mostly allopatric, and central plus southeastern Texas is the only region where we find them both.

However, it is conceivable that one of these two barcodes might have evolved not within this species, but could have been introgressed from a different, albeit closely related, species. If that were the case, it is possible that the two *Heraclides cresphontes*-like taxa are not distinct as species, but one simply experienced introgression from a different species. For instance, many individuals of *Erynnis propertius* (Scudder & Burgess, 1870) in California carry DNA barcode of *Erynnis horatius* (Scudder & Burgess, 1870) from eastern USA (Zakharov et al. 2009). To test whether the southwestern *Heraclides cresphontes*-like barcode sequence might have been introgressed from some other *Heraclides* species, instead evolving within *Heraclides cresphontes* populations, we obtained barcode sequences of eight *Heraclides homothoas* and five *Heraclides melonius* specimens from several localities (Suppl. material 1). These two taxa are from the same species group of Giant Swallowtails and are the closest extant relatives of *Heraclides cresphontes*. Sequences of *Heraclides homothoas* and *Heraclides melonius* were different from each other (2.6%) and from either of the *Heraclides cresphontes* DNA barcodes (3% to 3.5%, differences of the same magnitude as those between the two *Heraclides cresphontes*-like taxa). Barcode sequences were also obtained from all *Heraclides thoas* subspecies and from *Heraclides paeon*. These sequences were even more distant from the *H cresphontes* group (more than 5% difference), but revealed unexpected peculiarities of their own. Thus, neither *Heraclides cresphontes*-like sequence was introgressed from any extant *Heraclides* taxon, and the two sequences: eastern and southwestern, are each other’s closest relatives.

Next, in a quest for the names to apply to the eastern and southwestern species, we analyze names proposed for *Heraclides cresphontes*, search for type specimens, and stabilize nomenclature by designation of lectotypes and a neotype.

**Figures 1–6.**
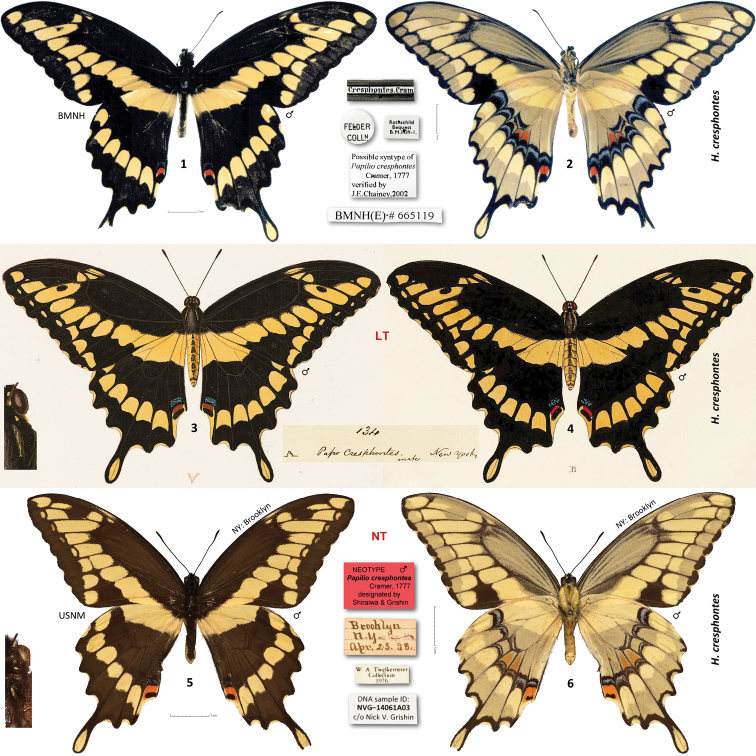
*Heraclides cresphontes* type specimens and illustrations. **1–2** possible paralectotype [BMNH] **3** Lambertz original illustration of the lectotype designated herein, specimen apparently lost **4** published engraving of the lectotype (Cramer, 1777) **5–6** neotype ♂ designated herein. Data in text and Supplementary Table 1. Dorsal/ventral surfaces are in odd/even-numbered figures, except 4, which shows dorsal. Labels are shown between the images of the same specimen, 3-fold magnified segment of head, neck and thorax is on the left and a 6-fold magnified dorsoposterior view of abdomen is on the right. 1 cm scale bars for specimens and labels are shown. Images 1–2 are by Gerardo Lamas, copyright Trustees of the Natural History Museum, London; used with permission.

### Lectotype designation for *Papilio cresphontes* Cramer, 1777

*Papilio cresphontes* was described by Pieter Cramer (1777: 106, 107, Pl. CLXV A, CLXVI B) on the basis of several specimens collected “in Noord-Amerika, te Nieuwjork en op het Eiland Jamaika, als mede in Zuid-Carolina” (description on the left in Dutch), or “dans l’Amerique Septentrionale, a la Nouvelle-York & dans l’Isle de la Jamaïque, comme aussi dans la Caroline Meridionale” (description on the right in French), which can be translated as “in North America, from New York and on the island of Jamaica, as well as in South Carolina.” Thus, the type locality includes at least three distinct localities in North America: New York, Jamaica, and South Carolina. However, the species referred to as *Heraclides cresphontes* in essentially all literature since Cramer does not normally occur in Jamaica, and the Jamaican records are either possible human imports or misidentifications (Tyler et al. 1994). The native Jamaican Giant Swallowtail has been described by Rothschild and Jordan (1906) as a different taxon, known today as *Heraclides melonius*. Rothschild and Jordan (1906) referred to Cramer’s description of *Heraclides cresphontes* in their description of *Heraclides melonius*, hence making the Jamaican syntype of *Heraclides cresphontes* simultaneously a syntype of *Heraclides melonius* and the type series of *Heraclides cresphontes* polytypic. Polytypic series of *Heraclides cresphontes* has a potential for instability of nomenclature in the absence of a lectotype. To designate the lectotype, we studied the *Heraclides cresphontes* type series further.

Cramer illustrated two specimens, a female in dorsal and ventral aspects (Cramer 1777: pl. CLXV A, ventral aspect figure erroneously referred to as “B” on page 107) and a male in dorsal aspect (Cramer 1777: pl. CLXVI B, page 107 states the ventral aspect of the male is similar to that of the female illustrated in pl. CLXV A). Published illustrations did not give precise localities of these specimens. Additionally, Cramer referenced a specimen illustrated by Daubenton (1765: 69). This specimen is equally a syntype. The engraving (Daubenton 1765: 69), masterfully performed by French artist Francois Nicolas Martinet (1725–1804) shows dorsal and ventral aspects of what was called “Le Festonne de la Gouadeloupe”. Apparently, “Le Festonne” (The Scalloped), is a name given to this butterfly, and “la Gouadeloupe” could only mean the locality, i.e., Guadeloupe, a group of Caribbean islands, which today are an overseas region of France. Curiously, this specimen is also a syntype of *Heraclides thoas* (Linnaeus, 1771), and its locality as “Guadalupa” is mentioned in the original description (Linnaeus 1771).

Since no Giant Swallowtails are known from Guadeloupe (Tyler et al. 1994), either this locality is erroneous, or the species illustrated has become extinct there. The engraving is consistent in most characters with the well-patterned spring form of eastern *Heraclides cresphontes*. However, the prominent red-orange base of all three cells between veins M_1_ and CuA_1_ on ventral hindwing is a character diagnostic of *Heraclides melonius*, currently known only from Jamaica. Thus, using published illustrations and texts we were not able to confidently associate any of the three illustrated syntypes with localities mentioned in the description of *Heraclides cresphontes*.

We took the following steps to search for the *Heraclides cresphontes* syntype specimens. First, we studied the literature. In a comprehensive search for the type specimens of Cramer taxa in the Natural History Museum, London (BMNH), John Chainey identified a possible syntype male (specimen number BMNH(E)#665119, Figs 1–2). Unfortunately, the specimen does not bear any locality labels. Also, it is not any of the three syntypes discussed above: it is a male and it lacks 3 anterior yellow spots on the dorsal forewing. Finally, as Chainey noticed, it is not possible to say with any confidence that the specimen is indeed a syntype. Second, we contacted Rob de Vos, Lepidoptera collection manager at Naturalis Biodiversity Center (Leiden, Netherlands), where Cramer types for a number of taxa are cared for. The search for *Heraclides cresphontes* syntypes in both collections—Rijksmuseum van Natuurlijke Historie (RMNH) and Zoölogisch Museum Amsterdam (ZMAN), did not yield positive results. Rob wrote: “unfortunately no luck for a Cramer’s cresphontes type. I’m afraid we do not have it. It may not exist anymore. Much of his collection has disappeared, probably lost, in private collections.” Thus we were not able to find possible syntypes with locality labels to help select a single locality of several mentioned in the description of *Heraclides cresphontes*.

Similar challenges with Cramer type localities and syntypes were encountered by other researchers, so negative results were not surprising. For instance, Clench and Miller (1980) found that “many of the species described by Cramer in *De Uitlandsche Kapellen* were based on specimens brought to him by seafarers, and Cramer accepted their locality labels as correct”. Clench and Miller also examined possible shipping routes seafarers have taken and listed Jamaica, New York, Savannah and Chesapeake Bay as one of those possible routes, consistent with all the localities given by Cramer.

As a final resort, we consulted the original drawings by Gerrit Wartenaar Lambertz made for Cramer and used as prototypes for the published engravings. Presently, the drawings are in the library of the Natural History Museum, London (BMNH). One of the drawings for the Volume 2, “A” on the fig #134, reproduced here as Fig. 3, was a prototype for Cramer’s figure CLXVI B, male from the original description, reproduced here as Fig. 4. Interestingly, the caption to the Lambertz figure stated “*Cresphontes male New York*” (inset between Figs 3 and 4). The image portrays a specimen very consistent with the spring form of eastern *Heraclides cresphontes*, with well-developed row of submarginal spots on the forewing above, rounded tails, and yellow spots on the neck, not joined into stripes (inset to Fig. 3). Although it is impossible to be certain that “New York”, as stated below the drawing, is indeed the true locality where this specimen was captured (Clench and Miller 1980), *Papilio cresphontes* has been reported from northern states such as New York prior to 1900s (Dwight 1882, Holland 1898), even if it was quite rare. Nevertheless, “New York” was the first of the three specific localities mentioned by Cramer in the *Heraclides cresphontes* description. Also, the male specimen illustrated is heavily patterned. Such specimens are less common in collections and may be easily recognizable if this syntype is eventually found. For these reasons, this male specimen, illustrated by Lambertz in Volume 2, fig 134, figure 3 and stated to be from “New York” and reproduced in Cramer (1777) fig CLXVI B, with a complete row of 7 submarginal spots on the forewing, small oval dark-brown spot in the anterobasal quadrant of the yellow spot in forewing cell R_5_-M_1_, and small yellow spots on the head above and behind the eyes and on the patagia, not connected to yellow lines on tegulae, is hereby designated the lectotype of *Papilio cresphontes* Cramer, 1777. The identity of the lectotype is in agreement with the usage of this name since it was proposed. The lectotype is designated to ensure nomenclatural stability due to polytypic type series including two species, and to clarify the type locality, which becomes USA: New York. We were not able to locate the lectotype and consider it to be lost.

### Lectotype designation for *Papilio thoas* Linnaeus, 1771

The above lectotype designation resolves the problem between *Heraclides cresphontes* and *Heraclides melonius*, both of which were in the *Heraclides cresphontes* type series; and for the interest of stability secures traditional usage of these names: *Heraclides cresphontes* for eastern North America populations, and *Heraclides melonius*, in accord with its original description (Rothschild and Jordan 1906), for Jamaica. Additionally, if a *Heraclides cresphontes* syntype is found, unless there is strong evidence that it is the specimen pictured on the Cramer fig CLXVI B (and the original Lambertz illustration shows excellent details), it is not a name bearing type. The next problem is that the *Heraclides thoas* type series included at least one *Heraclides cresphontes* specimen, the one from Daubenton (1765: 69), discussed above. Due to the inclusion of this specimen in the original description by Linnaeus (1771) and a type locality originally listed as “Guadalupa, Surinamo”, a potential for nomenclatural instability exists. Rothschild and Jordan (1906) considered the Daubenton illustration, which locality “Guadalupa” refers to, to be *Heraclides cresphontes*. Honey and Scoble (2001) concurred with this conclusion, stating: “the type locality of *thoas* can, with good reason, be restricted to Surinam”, but they didn’t designate the lectotype. Linnaeus (1771) listed specimen(s) illustrated by Drury (1770: pl. 22, figs 1, 2) first, before listing other references. The Drury illustrations are excellent and agree with the subsequent usage of the name, and the locality is stated in the text: “Surinam” (Drury 1770: 45). To stabilize nomenclature and clarify the type locality, we designate a specimen with 4 submarginal yellow spots, a small spot near the apex of the dorsal forewing and a yellow spot at the posterodistal end of the forewing discal cell, illustrated in Fig. 1 on the Drury (1770) Plate 22 as the lectotype of *Papilio thoas* Linnaeus, 1771. The whereabouts of the lectotype are unknown, but the Drury illustration is of sufficient quality to confidently identify the species and see that the lectotype is in agreement with the prior and current usage of the name. The type locality of *Heraclides thoas* becomes Suriname.

### Neotype designation for *Papilio cresphontes* Cramer, 1777

The two lectotypes designated above unambiguously distinguish *Heraclides cresphontes*, an eastern US species, from *Heraclides melonius* and *Heraclides thoas*. However, with our finding that *Heraclides cresphontes* is a complex of two sister species, the lectotype, which is apparently lost, may not be sufficient to define the taxon from just a single illustration. While most specimens of the southwestern species differ from the eastern *Papilio cresphontes* in neck and wing patterns, its diagnostic characters are found in genitalia and DNA, and thus cannot be confirmed from the illustration alone. Therefore, we proceed with the designation of the neotype in accord with ICZN Article 75.3 (ICZN 1999). The exceptional need for the *Heraclides cresphontes* neotype arises to unambiguously distinguish it from the southwestern species, and have an actual name-bearing type specimen for future DNA research. A male specimen in the National Museum of Natural History, Smithsonian Institution, Washington, DC (USNM) mounted ventral side up, illustrated in Figs 5–6, and bearing the following three rectangular labels: faded to wheat color, handwritten - || Brooklyn | N. Y. ~ | Apr. 23. 98. ||; white printed - || W. A. Twelkemeier | Collection | 1976 ||; white printed - || DNA sample ID: | NVG-14061A03 | c/o Nick V. Grishin || is hereby designated as the neotype of *Papilio cresphontes* Cramer, 1777. The red printed label - || NEOTYPE ♂ | *Papilio cresphontes* | Cramer, 1777 | designated by | Shiraiwa & Grishin || will be added to the neotype upon publication of this study. Length of the neotype forewing is 48 mm, and this specimen can be recognized by a unique pattern of minor damage along the anal margin of left hindwing basad of the blue crescent, and missing tip of the tail on right hindwing (Fig. 5). Neotype is an old specimen, collected in 1898, although over a century more recent than the Cramer’s type, it may still better represent the fauna of New York prior to major industrial developments. According to ICZN Code Art.76.3., the type locality of *Heraclides cresphontes* becomes USA: New York, Brooklyn.

This neotype designation satisfies all seven provisions of the ICZN code (Art. 75.3.1.–7.) as follows. The neotype is designated to clarify the attribution of the name *Heraclides cresphontes* to the eastern (and not southwestern) species in accord with traditional usage of the name and the type locality of the lectotype (Art. 75.3.1.) The characters to differentiate *Heraclides cresphontes* from the southwestern species are listed in the first two paragraphs of the Results section and additionally in the Diagnosis section of the description below (Art. 75.3.2.). The neotype and its labels are shown in Figs 5–6 (Art. 75.3.3.). The steps taken to trace the lectotype are described above (Art. 75.3.4.). Neotype agrees with the original description and is rather similar to the lectotype illustration: it is of a broadly patterned yellow spring form, with all 7 submarginal yellow spots on the forewing expressed, and with small yellow spots on head, patagia, and tegulae (Art. 75.3.5.). The lectotype was stated to be from “New York”, and the neotype was collected in Brooklyn, New York, closely matching the locality of the lectotype (Art. 75.3.6.). The neotype is in the National Museum of Natural History, Smithsonian Institution, Washington, DC (USNM) (Art. 75.3.7.). Facies, DNA barcode ID tag (GenBank Accession KP173859), and locality of the neotype unambiguously attribute the name *Heraclides cresphontes* to the eastern species, leaving the southwestern species for further analysis.

### Analysis of names proposed for *Heraclides cresphontes*

Eight names have been considered synonyms of *Heraclides cresphontes* by Lamas (2004) and Pelham (2008). Out of these, *Heraclides oxilus* Hübner, [1819] is an objective junior synonym, because it was proposed as a replacement name for *Heraclides cresphontes*, erroneously considered to be preoccupied; Papilio cresphontes var. maxwelli Franck, 1919 and *Papilio cresphontes pennsylvanicus* F. Chermock & R. Chermock, 1945 are subjective junior synonyms, based on specimens from USA: Florida: Pinellas County, St. Petersburg (Franck 1919b) and USA: Pennsylvania, Centre County, State College, respectively.

We consider *maxwelli* to be an available name according to ICZN Code Art. 45.6.4., because (1) it was published before 1961, (2) the author used the terms “var.” and “variety”, and (3) the publication does not unambiguously reveal that the name is infrasubspecific. The entire description text is very short, cited here: “The triangular spot near the apex of the primaries is entirely filled out with sulphur yellow, giving the specimen a striking tropical appearance. This variety is named after my esteemed friend Mrs. J. B. Maxwell, of Faribault, Minn.” (Franck 1919a). The *maxwelli* holotype (“the specimen”) is a strongly patterned with yellow, mostly likely spring brood individual, probably somewhat aberrant. It is eastern *Heraclides cresphontes* by facies, and is pictured in Warren et al. (2014). We agree with Lamas (2004) and Pelham (2008) in treating the name as a synonym of *Heraclides cresphontes*, because we do not see consistent differences between Florida populations of *Heraclides cresphontes* and those from northeastern US near the *Heraclides cresphontes* type locality.

The name *pennsylvanicus* was proposed as a subspecies (Chermock and Chermock 1945). The type series specimens are characterized by weaker developed dark pattern on ventral side of wings, suggesting that specimens from the northeastern parts of the range may be weaker patterned in black. Facies of the *Heraclides cresphontes pennsylvanicus* holotype and facies and DNA barcodes of two paratypes from the same locality are of *Heraclides cresphontes* and not of the southwestern species. However, paler specimens are found throughout the range of *Heraclides cresphontes*, not only in the northeast, and are occasional even in the southwestern *Heraclides cresphontes*-like species. *Heraclides cresphontes* neotype is also from northwest and is weaker patterned with black below (Fig. 6). Therefore, we concur with Pelham (2008) that it is best to treat *pennsylvanicus* as a subjective junior synonym of *Heraclides cresphontes* rather than a meaningful subspecies.

The remaining six names are infrasubspecific according to the Articles 45.5. & 45.6. of the ICZN Code, in agreement with Pelham (2008) and therefore are unavailable. Three were proposed explicitly for aberrations: ab. (nov.) *lurida* Schultz, 1908; ab. *luxuriosa* Reiff, 1911; and ab. *intacta* Strand, 1918, and thus are infrasubspecific according to Art. 45.6.2. For the final two names, both published before 1961: tr. f. *forsythae* Gunder, 1933; and forma *melanurus* Hoffmann, 1940, “the content of the work unambiguously reveals that the name was proposed for an infrasubspecific entity” (ICZN 1999: Art. 45.6.4.). The first one was based on specimens from USA: Florida and the facies of the holotype agree with *Heraclides cresphontes*.

However, the second one, Papilio cresphontes forma melanurus, is from Mexico: Guerrero, and the facies of the holotype imply that it is the southwestern species, not *Heraclides cresphontes*. Although its name as originally proposed contains the word “forma”, the text of the description (Hoffmann 1940) is clear about it being a form with dark tails inside populations of typical *Heraclides cresphontes* with yellow-spotted tails: “It differs from cresphontes cresphontes Cramer by its entirely black tails that lack the typical yellow spot. The form is found together with typical cresphontes with some frequency in the Balsas River basin and the mountains of the State of Guerrero” (translated from Spanish original)—i.e., “La forma se encuentra junto con cresphontes typicus con cierta frecuencia” unambiguously implies that the name is infrasubspecific: black-tailed *Heraclides cresphontes* that flies together with typical *Heraclides cresphontes* in Guerrero is not its subspecies, but a form. From the description, it is equally unambiguous that Hoffmann considered populations in Mexico: Guerrero to be typical *Heraclides cresphontes*. The name *melanurus* was not adapted for a subspecies since it was proposed. Therefore, the southwestern *Heraclides cresphontes*-like species does not have a name and is named herein.

#### Heraclides rumiko

Taxon classificationAnimaliaLepidopteraPapilionidae

Shiraiwa & Grishin sp. n.

http://zoobank.org/F876D822-AB4D-44DF-AB7C-02C5131B91FA

**Figures 7–10.**
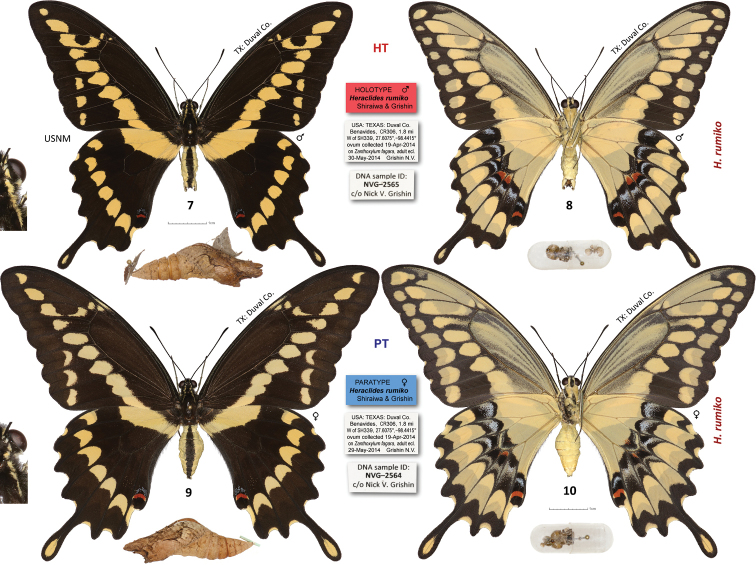
*Heraclides rumiko* type specimens: **7–8** holotype ♂ **9–10** paratype ♀ NVG-2564, data in text and Supplementary Table 1. Dorsal/ventral surfaces are in odd/even-numbered figures. Labels are shown between the images of the same specimen, exuvia and head capsules in a gelatin capsule are below, and 3-fold magnified segment of head, neck and thorax is on the left. All images are to scale (including labels), except the magnified insets.

**Figure 11.**
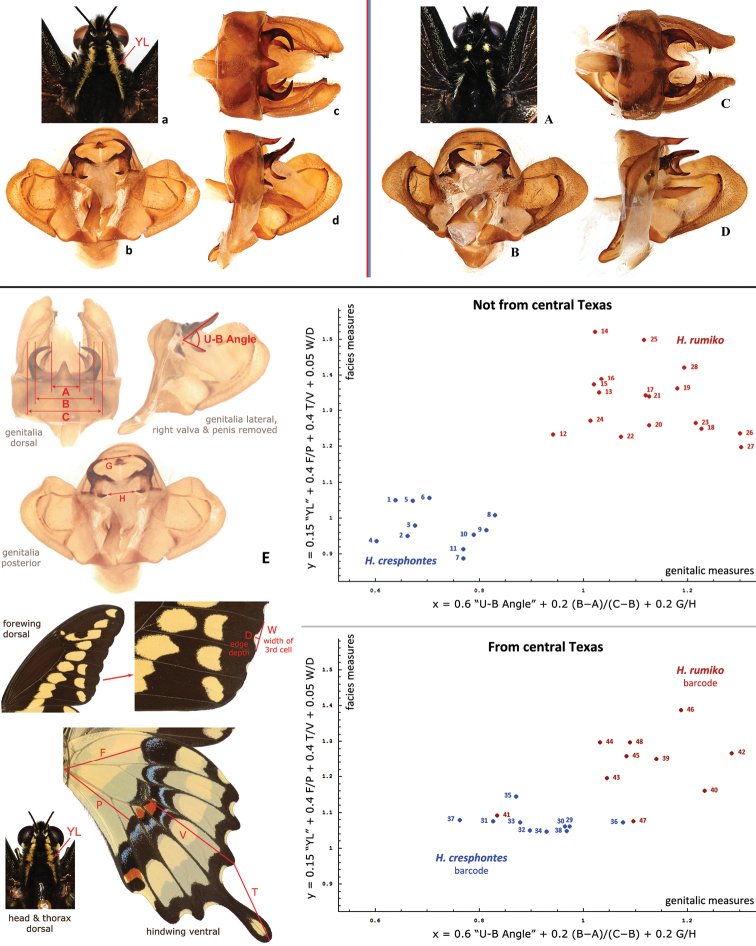
Neck pattern, male genitalia, and morphometrics. **a**–**d** *Heraclides rumiko*, paratype, Mexico: Baja California Sur: Buena Vista, 1-Oct-1981, leg. D. Faulkner & F. Andrews, genitalia KS017 [SDMC] **A–D** *Heraclides cresphontes*, USA: Georgia: Clark Co. July 2009, genitalia KS009 **E** Morphometric measurements performed on genitalia and facies and plotted in two dimensions. Horizontal axis is a weighted average of the three genitalic measures: 0.6*”U-B Angle” + 0.2* (B−A)/(C−B) + 0.2*G/H. “U-B Angle” is measured in radians. Vertical axis is a weighted average of the four facies measures: 0.15*”YL” + 0.4*F/P + 0.4*T/V + 0.05 W/D, where “YL” is equal to 0 or 1, if yellow line on the neck is separated into spots or continuous, respectively. Measured distances are indicated on the illustrations. Each of the two (genitalic and facies) linear combinations of measures completely segregates *Heraclides rumiko* (red points) from *Heraclides cresphontes* (blue points) specimens (not from central Texas) with a hiatus. Even a single measure “U-B Angle” identifies all specimens correctly, except #12, which has a brachium strongly curved dorsad. Specimen localities: *Heraclides cresphontes*: 1. GA: Clark Co.; 2. NY: Niagra Co., Lockport; 3. NC: Carteret Co.; 4. IN: Kosciusko Co., Silver Lake; 5. WI: Sauk Co., Sauk City; 6. LA: St. John Pa., Edgard; 7. AR: Osceola; 8. OK: Marshall Co., Lake Texoma; 9. FL: Okeechobee Co., Fort Drum; 10. OH: Montgomery Co., Dayton; 11. PA: York Co., Pinchot State Park. *Heraclides rumiko*: 12. AZ: Maricopa Co., North Phoenix; 13. AZ: Santa Cruz Co., Sycamore Canyon; 14. CA: Imperial Co.; 15. MX: Veracruz, Fortin de las Flores; 16. MX: Oaxaca, Yangul; 17. Costa Rica: Puntarenas, San Antonio; 18. MX: Tamaulipas, Gomez Farias; 19. MX: Colima, Colima; 20. MX: Sonora; 21. MX: Yukatan, Merida; 22. MX: Morelos, Rancho Viejo; 23. MX: BCS, Buena Vista; 24. MX: Jalisco, Ajajic; 25. CA: San Diego Co., La Jolla; 26. MX: Quintana Roo, nr. X-Can; 27. TX: Val Verde Co., Del Rio; 28. CA: San Diego Co., Pauma Valley. Central Texas specimens are from Bexar (33–38, 42–48), Williamson (39–41), Travis (31, 32), Bastrop (30), and Brazos (29) Counties. Voucher codes for these specimens are: 29. NVG-2236; 30. -2299; 31. -2300; 32. -2174; 33. -2192; 34. -2196; 35. -2205; 36. -2209; 37. -2210; 38. -2216; 39. -2301; 40. -2225; 41. -2229; 42. -2191; 43. -2197; 44. -2204; 45. -2208; 46. -2211; 47. -2215; 48. -2218. Species (color on the plot) is assigned to central Texas specimens by COI barcode. See Supplementary Table 1 for more data. Specimens 36 and 41 are apparent hybrids or the results of introgression.

**Figure 12.**
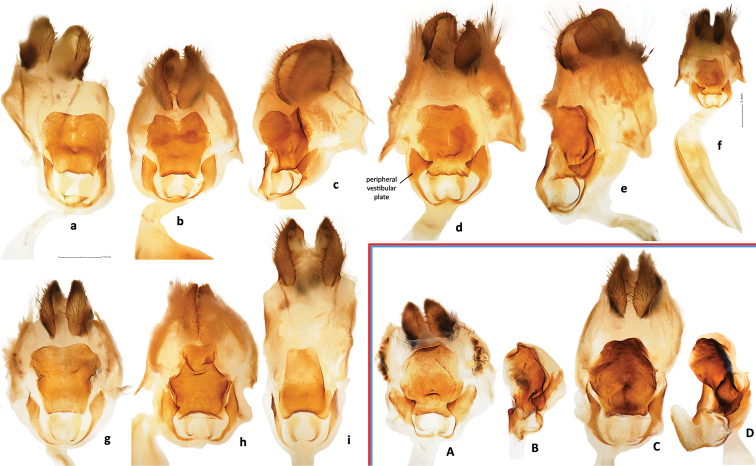
Female genitalia. **a**–**i** *Heraclides rumiko* paratypes [TAMU]: **a** USA: TX: Cameron Co., Las Palomas WMA, Tucker Unit, 24-Oct-2001, leg. J. & F. Preston, DNA voucher NVG-2238, genitalia NVG140320-79 **b**, **c** USA: TX: Hidalgo Co., Santa Ana National Wildlife Refuge, 13-Oct-1968, leg. R. O. Kendall & C. A. Kendall, NVG-2163 | NVG140320-04 **d**–**f** USA: TX: Cameron Co., World Wildlife Management Area nr. Santa Maria, 14-Nov-1971, leg. R. O. Kendall & C. A. Kendall, NVG-2195 | NVG140320-36 **g** Honduras: Escuela Agrícola Panamericana, 30 km SE Tegucigalpa, 1-May-1985, leg. Vascones, NVG-2221 | NVG140320-62 **h** Mexico: Durango: Tlahualilo, 20-Aug-1935, leg. C. S. Rude, NVG-2230 | NVG140320-71; **i** Mexico: Tamaulipas: Cd. Monte, Los Arcos Ct., 8-May-1978, leg. R. O. Kendall & C. A. Kendall, NVG-2185 | NVG140320-26 **A**–**D** *Heraclides cresphontes*, USA: MO: **A**, **B** Phelps Co., Mark Twain National Forest, DeWitt Pond, N37.8367 W91.9385, 25-May-2006, J. C. Abbott, NVG-2293 | NVG140403-21 [TMMC] **C**, **D** Montgomery Co., NVG-2242 | NVG140320-83 [TAMU]. Ventrolateral view is shown in **c**, **e**, **B**, **D** (**e** is left-right inverted), others are in ventral view. All images are to scale shown under **a**, except **f**, which is half the size with scale shown to the right.

Figs 7–10 , 11a–d , 12a–i , 12E part, 13 part, 14 part, 15 part, 16 part, 17 part, 18 part, 19a–x, 20a–r, 21a–n, 22a–j, 23a–l

##### Description.

**Figure 13.**
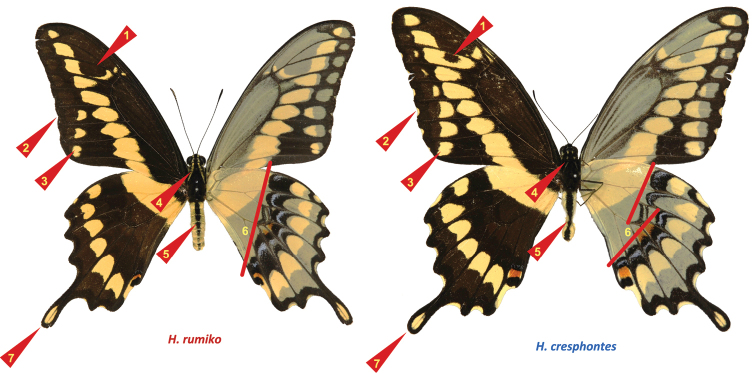
Facies differences between *Heraclides rumiko* (left, r) *Heraclides cresphontes* (right, c) indicated by red triangles and lines. These differences are as follows. **1)** Dark spot on forewing: (r) almost always large; (c) variable, but often weak and sometimes absent **2)** Forewing margin: (r) often straight with smaller or absent marginal spots; (c) strongly scalloped with yellow marginal spots at dips between veins **3)** Forewing submarginal yellow spots: (r) smaller rarely more than three; (c) frequently larger, more than three **4)** Thorax with: (r) yellow line running from head through patagia to tegulae; (c) spots instead of the line, or just few yellow scales. **5)** Abdomen: (r) usually with a fainter dark band; (c) often with solid dark band **6)** Inner edge of black discal band on ventral hindwing: (r) mostly straight; (c) usually curved **7)** Tail: (r) mostly narrow and relatively longer; (c) typically rounder and wide, shorter. *Heraclides rumiko* is usually smaller than *Heraclides cresphontes*, despite being a southern taxon. Due to significant seasonal and individual variation, none of these characters is fully reliable and exceptions exist. The head-neck-thorax line vs. spots (Fig. 11a, A) might be the strongest single character. A combination of characters should be used for reliable identification, e.g., the one shown in Fig. 11E. Many specimens in central Texas exhibit intermediate characters, atypical character combinations, and possible hybrids can be found (Fig. 11E).

Male (n=95, Figs 7–8, 11a, 13 part) – holotype forewing length = 50 mm. Size on average smaller (mean forewing length 54 mm, maximum observed 58 mm) than *Heraclides cresphontes*. Wings typically narrower, less scalloped, and wing shape less variable than in *Heraclides cresphontes*, hindwing tail longer and narrower, weakly spoon-shaped. Ground color black to dark chocolate-brown. **Dorsal forewing:** Two maize-yellow bands: a central band of 9 spots from apex to basal third at inner margin in all cells from R_3_-R_4_ to 1A; and a sub-marginal band of 3 to 7 spots in cells from R_4_-R_5_ to CuA_2_-1A, absent or vestigial in most specimens anteriad of three cells between M_3_ and 1A veins. Several smaller maize-yellow spots near costa at the end of discal cell. Background-colored dark oval spot of variable size inside or at the anterior edge of the yellow central band spot in cell R_5_-M_1_, sometimes dividing the yellow spot into two. Marginal pale spots at dips between veins small or almost absent. **Dorsal hindwing:** Two maize-yellow bands extending from forewing: undivided into spots central band in wing basal third from costa to inner margin, with small tooth-like protrusions distad along veins Rs and M_1_; and sub-marginal band of 7 spots in cells from Sc+R_1_-Rs to CuA_2_-1A+2A, the tornal spots up to margin. Maroon-red to orange-red eyespot near tornus with blue crescent above. Center of the tail tip yellow. **Ventral forewing:** Yellow color paler; wider yellow central band weakly divided into spots from forewing costa to inner margin; submarginal band of 8 or 9 (spot near tornus may be divided into two) spots larger than on dorsal side in cells between veins R_3_ and 1A. Marginal pale spots at dips between veins larger than above. Discal cell yellow, overscaled with dark and with 5 variously developed dark longitudinal streaks. **Ventral hindwing:** Largely maize-yellow, a dark-brown rather straight discal band from costa through the end of discal cell to tornus with blue crescents inside in each cell and orange-red tornal spot distal to blue crescent. Distal end of discal cell with black (in some specimens blue) lines, often fused with the median band. Cells M_2_-M_3_ and M_3_-CuA_1_ orange-red at the base; few or no orange-red scales at the base of M_1_-M_2_ cell. Margin bordered black, with yellow edges along concavities. Dark-brown rays along veins between discal and marginal dark bands. Tail tip with yellow spot in the middle. **Head and body:** Antennae dark-brown, segments ringed with yellow beneath. Head and thorax dark-brown dorsally and yellow ventrally. Two longitudinal yellow stripes on head, patagia and tegulae forming two continuous yellow lines from head to thorax (Fig. 11a), only rarely and weakly separated into spots. Abdomen yellow, with a black dorsal stripe fading posteriad in many specimens. **Male genitalia** (n=34; Fig. 11a–d, 14 part): Pseuduncus shaped as a tooth like, pointed projection flattened at the tip not extending posteriad beyond uncus, thus leaving a gap between the last tergum and valvae. In lateral view, pseuduncus dorsally flat but ventrally convex towards the tip. Uncus more slender than in *Heraclides cresphontes*, divided into two curved horn-like arms; each arm directed posterodorsad, curved laterad initially and then strongly mediad, narrowing to a point. Brachium arms from the base of uncus ventrad, narrow, shorter than uncus, directed posteroventrad and mediad, differently from uncus in dorsoventral projection. Both uncus and brachium visible in dorsal view (Fig. 11c). In *Heraclides cresphontes* brachium mostly covered by uncus (Fig. 11C). Valva somewhat square in shape, broadly rounded at the angles. Harpe oval, without long projections and spikes, finely dentate ventrad in posterior half, with apex curved inward. Distal end of harpe very close to the edge of valva, closer than in *Heraclides cresphontes*, and valva projects distad from the denticulate edge of harpe less than in *Heraclides cresphontes*, costa of valva usually broader than in *Heraclides cresphontes*. Aedeagus as long as the valva, straight and stout, no cornuti. Juxta U-shaped, gracile and smooth. Saccus short, barely protruding anteriad beyond vinculum.

Female (n=28, Figs 9–10): Similar to male but larger, with broader wings, ground color paler, yellow bands typically narrower and paler: cream-yellow on forewing and somewhat yellower on hindwing. **Female genitalia** (n=11, Fig. 12a–i): Lamella postvaginalis tongue-shaped, ventrally convex smooth fig somewhat longer than wide, with rounded or slightly concave posterior margin, variable in width and length, lamella antevaginalis narrow, poorly sclerotized, laterally extending into narrow peripheral vestibular figs surrounding lamella postvaginalis on the sides up to its middle. Inner edge of each fig with short, tooth-like projection in some specimens (Fig. 12e). Antrum with two weakly sclerotized small figs along sides. Ductus bursae short, not longer than sterigma. Corpus bursae with a long longitudinal signum on ventral side.

**Figure 14.**
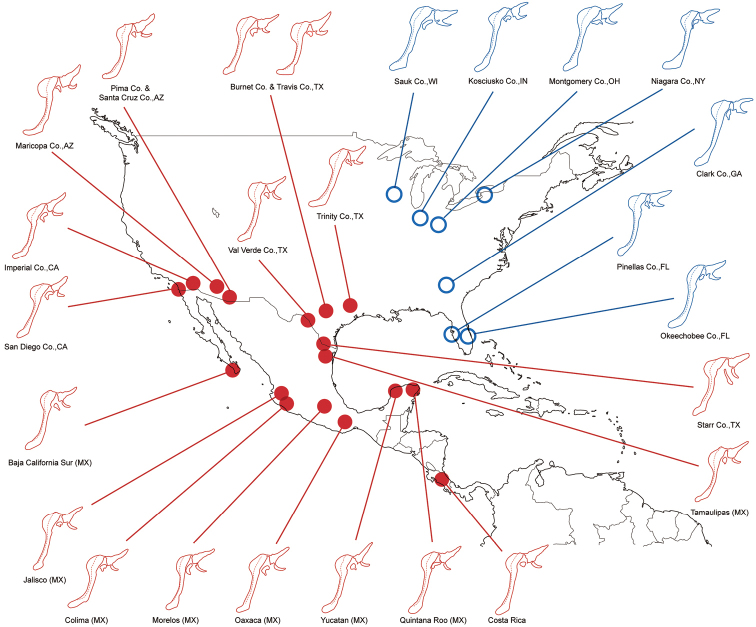
Variation in male genitalia. Left lateral view of genital ring (uncus, brachium, dorsolateral sclerite, tegumen, vinculum and saccus) is shown, valvae, aedeagus and last tergum with pseuduncus are removed. *Heraclides cresphontes* and *Heraclides rumiko* localities are shown in blue circles and red disks, respectively.

##### Barcode sequence of the holotype.

Genbank accession KP173713, 658 base pairs:

AACATTATATTTTATTTTTGGAATTTGAGCAAGAATACTAGGAACTTCTCTTAGTTTACTAATTCGTACTGAATTAGGCA CCCCCGGCTCATTAATTGGAGATGATCAAATTTATAATACTATTGTTACAGCTCATGCTTTTATTATAATTTTTTTTATAG TTATACCTATTATAATTGGAGGATTTGGAAATTGATTAATTCCATTAATATTAGGAGCCCCTGATATAGCTTTTCCTCGTA TAAATAATATAAGATTTTGACTTTTACCCCCTTCTCTAACTCTCCTAATTTCAAGAATAATTGTAGAAAATGGGGCAGGAA CTGGATGAACTGTTTACCCTCCTCTTTCCTCTAATATTGCCCATGGAAGAAGATCAGTAGATTTAGTTATCTTTTCTTTAC ATTTAGCTGGTATTTCCTCAATTCTTGGAGCAATTAATTTTATTACTACAATTATTAATATACGAATTAATAGAATATCTT TTGATCAAATACCTTTATTTGTTTGAGCCGTAGGAATTACAGCTTTATTATTACTTTTATCTTTACCTGTTTTAGCAGGAG CTATTACTATACTTTTAACTGATCGAAATTTAAATACTTCATTTTTTGACCCTGCTGGAGGAGGAGATCCAATTTTATACC AACATTTATTT

In addition to the holotype, barcodes and ID tags were obtained for 110 paratypes: 93 full-length barcodes (658 to 664 bp), 3 partial barcodes (443 bp) and 14 ID tags (64 bp), see Suppl. material 1, GenBank accessions: KP173714–KP173823. Full length barcodes of paratypes revealed ten haplotypes differing from each other by just 1 to 3 base pairs (less than 0.5%, Fig. 17). The haplotype of the holotype was most frequently observed (67 sequences).

##### Type material.

**Holotype:** ♂, has the following three rectangular labels: white printed - || USA: TEXAS: Duval Co. | Benavides, CR306, 1.8 mi | W of SH339, 27.6075° −98.4415° | ovum collected 19-Apr-2014 | on *Zanthoxylum fagara*, adult ecl. | 30-May-2014 Grishin N.V. ||; white printed - || DNA sample ID: | NVG-2565 | c/o Nick V. Grishin ||; red printed - || HOLOTYPE ♂ | *Heraclides rumiko* Shiraiwa & Grishin ||. Pupal exuvia and larval head capsules are stored with the holotype. The holotype is illustrated in Figs 7–8, 19a–d, v, & 23a–c, and the Genbank accession for its DNA COI barcode sequence is KP173713. Upon publication, the holotype will be deposited in the National Museum of Natural History, Smithsonian Institution, Washington, DC (USNM). **Paratypes:** 94 ♂♂ and 28 ♀♀. Of these, 1 ♂ and 2 ♀♀ with the same data as the holotype, eclosion dates 26-, 27-, 29-May-14; DNA vouchers NVG-2559, -2563, -2564. **USA: Texas:** 28 ♂♂ and 6 ♀♀ Duval Co., along SH339, 1–5 mi SW Benavides, 19-Apr-2014, leg. N. V. Grishin & Q. Cong, DNA vouchers NVG-2335–-2339, -2425–-2453; Duval Co., SH359, 7.6 mi NE Benavides, 27°40.443’ -98°19.209’, ex larva, leg. N. V. Grishin, eclosed: 1 ♂ and 1 ♀ 16-Nov-2003, NVG-38, -2097; 1 ♂ 25-Nov-2003, leg. N. V. Grishin, NVG-2095; 2 ♂♂ 3-Jun-2004, leg. N. V. Grishin, NVG-2098, -2099; Cameron Co., E of Brownsville, Palmetto Hill Rd., leg. N. V. Grishin: 1 ♂ 11-Nov-1996, NVG-2102; ex larva, 1 ♀ 5-Dec-2007, NVG-2100; 1 ♀ Cameron Co., World Wildlife Management Area nr. Santa Maria, 14-Nov-1971, leg. R. O. Kendall & C. A. Kendall, NVG-2195 | NVG140320-36 [TAMU]; 1 ♀ Cameron Co., Las Palomas WMA, Tucker Unit, 24-Oct-2001, leg. J. & F. Preston, NVG-2238 | NVG140320-79; 1 ♂ Cameron Co., Brownsville, 1-Apr-1981, leg. C. Bordelon, NVG-2223 | NVG140320-64 [TAMU]; Hidalgo Co., Mission, 10th St. at irrigation ditch [TAMU]: 1 ♂ 2-Sep-1972, leg. W. W. McGuire, NVG-2164 | NVG140320-05; 1 ♂ 1 ♀ 8-Sep-1972, leg. R. O. Kendall & C. A. Kendall, NVG-2165, -2166 | NVG140320-06, -07; 2 ♂♂ Hidalgo Co., McAllen [TAMU]: 9-Oct-1973, leg. W. W. McGuire, NVG-2168 | NVG140320-09 [TAMU]; Valencia Motel, ex larva 21-Oct-1972, larval foodplant *Ptelea trifoliata*, leg. R. O. Kendall & C. A. Kendall, NVG-2167 | NVG140320-08 [TAMU]; 1 ♀ Hidalgo Co., Santa Ana National Wildlife Refuge, near Alamo, 13-Oct-1968, leg. R. O. Kendall & C. A. Kendall, NVG-2163 | NVG140320-04 [TAMU]; 1 ♂ San Patricio Co., SH 77 ca. 7 mi NNE of Sinton, Welder Wildlife Foundation, 10-Aug-1968, leg. R. O. Kendall & C. A. Kendall, NVG-2173 | NVG140320-14 [TAMU]; 1 ♀ San Patricio Co., 12.5 km NE Sinton @ SH 77, Welder Wildlife Foundation, 28.113, -97.418, 1–3-Jul-2002, J. C. Abbott & Field Entomology Class, NVG-2302 | NVG140403-30 [TMMC]; 1 ♂ Refugio Co., US Hwy 77 Mission River SW of Refugio, ex larva 17-Nov-1963, larval foodplant *Zanthoxylum fagara*, leg. R. O. Kendall & C. A. Kendall, NVG-2171 | NVG140320-12 [TAMU]; 1 ♂ Brazos Co., College Station, Riley Estate, 30.58849, -96.25366, 14–15-May-2011, leg. M. L. Riley, NVG-2243 | NVG140320-84 [Ed Riley]; 1 ♂ La Salle Co., 10.1 mi NW Artesia Wells, Chaparral Wildlife Management Area, 11–15-Jun-2001, J. C. Abbott & Field Entomology Class, NVG-2298 | NVG140403-26 [TMMC]; 1 ♂ Kinney Co., 7 mi along railroad W of Spofford, 8-Oct-1966, leg. R. O. Kendall & C. A. Kendall, NVG-2169 | NVG140320-10 [TAMU]; Val Verde Co., Old US90 at Devils River W of Del Rio, ex larva larval foodplant *Ptelea trifoliata*, leg. R. O. Kendall & C. A. Kendall [TAMU]: 1 ♂ 16-Sep-1968, NVG-2176 | NVG140320-17; 1 ♀ 8-Jun-1968 NVG-2175 | NVG140320-16; 1 ♂ Val Verde Co., Seminole Canyon State Historic Site, 26-May-2007, 29.696, -101.336, leg. J. C. Abbott & Field Entomology Class, NVG-2289 | NVG140403-17 [TMMC]; 1 ♂ Terrel Co, 15 air mi S Sheffield, Oasis Ranch, Independence Cr., 30.467, -101.801, 595 m, 23–27-May-2007, J. C. Abbott & Field Entomology Class, NVG-2290 | NVG140403-18 [TMMC]; 1 ♀ TX: Tom Green Co., San Angelo, 13-Jul-1986, leg. P. Goroy, NVG-14081G05 [CSUC]; 1 ♀ Kerr Co., Kerrville, Riverside Nature Center, 10-Oct-1998, leg. W. F. Chamberlain, NVG-2226 | NVG140320-67 [TAMU]; 1 ♂ Kimble Co., Junction, 9-Oct-1966, leg. W. F. Chamberlain, NVG-2231 | NVG140320-72 [TAMU]; 1 ♀ Van Zandt Co., 2.5 mi W Van, IH20 rest stop, 28-Jul-1996, leg. N. V. Grishin, NVG-2092; Culberson Co., Guadalupe Mnts. National Park [CSUC], 1 ♂ Choza Spr., 5000’, 5-Jul-1986, leg. R. W. Holland & S. J. Cary, NVG-14081G12; 1 ♀ Pine Spring Cmpgr., 5700’, 20-Jun-1986, leg. R. W. Holland, NVG-14081G11. **USA: Colorado:** 1 ♂ Logan Co., Sterling, 10-Jul-1970, leg. D. L. Munget, NVG-14081G09 [CSUC]. **USA: New Mexico:** 5 ♂♂ [CSUC]: Torrance Co., Manzano Mts., 3 mi E of New Canyon Cmpgr. 7400’, 30-Jul-1968, leg. R. W. Holland, NVG-14081H02; Eddy Co., Last Chance Canyon, Sitting Bull Falls, 4600’, 2-Sep-1986, leg. R. W. Holland & S. J. Cary, NVG-14081H09; Eddy Co., above Sitting Bull Falls, 4600’, 20-Sep-1986, leg. R. W. Holland, NVG-14081H04; Eddy Co., Black River nr. Rattlesnake Spr., 3300’, 19-Jul-1986, leg. R. W. Holland, NVG-14081H01; Hidalgo Co., Peloncillo Mts., Guadalupe Canyon, SW slope, 4600’, 8-Sep-1985, leg. S. J. Carry, NVG-14081H05. **USA: Arizona:** 1 ♂ Maricopa Co., North Phoenix, 10-Aug-1974, | KS032; 1 ♂ Santa Cruz Co., Sycamore Canyon, 28-Aug-1977, leg. J. P. Brock, NVG-2109. **USA: California:** San Diego Co.: 1 ♀ San Diego, ex ovum 23-Sep-2012, eclosed 6-Nov-2012, leg. K. Shiraiwa, NVG-2505; 1 ♂ 1 ♀ Pauma Valley, 17-Jul-2011 & 25-Jun-2007, leg. K. Shiraiwa, NVG-2504, -2503; 1 ♂ 6762 Avenida Andorra, La Jolla, 2-Oct-1993, leg. J. Stoddard, | KS016; 1 ♂ Imperial Co., ovum collected 17-Apr-1962, adult eclosed 13-Jul-1962, | KS029. **Mexico: Baja California Sur:** 1 ♂ La Paz, Hotel Posada, 27-Sep-1970, leg R. W. Holland, NVG-14081G06 [CSUC]; 1 ♀ 2 mi N of San Pedro, 500’, 27-Sep-1970, leg. R. W. Holland, NVG-14081G04 [CSUC]; 3 ♂♂ Buena Vista [SDMC], 1-Oct-1981, leg. D. Faulkner & F. Andrews, NVG-2572– -2574; 1 ♂ ibid, | KS017; 1 ♂ 48 mi S La Paz, 25-Aug-1982, leg. D. Faulkner & J. Brown, NVG-2575. **Mexico: Sonora:** 1 ♂ 2 mi E San Carlos, 29-Jan-2003, leg. P. Opler, NVG-14081D10 [CSUC]; 1 ♀ Sonora, 5mi S of Yecora, 11-Aug-1991, NVG-2110; 1 ♂ Tepoca, 17-Sep-2010, | KS035 [SDMC]. **Mexico: Sinaloa:** 1 ♂ Panuco Rd. off Mx Hwy 40, 800’, 29-Nov-3-Dec-2002, leg. Opler & Buckner, NVG-14081E04 [CSUC]; 1 ♂ Mexico: Sinaloa, Mx Hwy 40, nr. Jct. of Mx 15, 400’, 30-Aug-1967, leg. R. W. Holland, NVG-14081H08 [CSUC]; 1 ♂ Mazatlan, 29-Dec-1974, NVG-2111. **Mexico: Colima:** 1 ♂ Colima, 5-Apr-1980, leg. P. Spade, | KS018 [SDMC]. **Mexico: Durango:** Tlahualilo, leg. C. S. Rude [TAMU]: 1 ♂ 19-Jul-1934, NVG-2232 | NVG140320-73, 1 ♀ 20-Aug-1935, NVG-2230 | NVG140320-71. **Mexico: Coahuila:** 1 ♂ Jct Hwy 57 & 53 south of Moncloya, 14-Sep-1977, leg. R. O. Kendall & C. A. Kendall, NVG-2180 | NVG140320-21 [TAMU]; 1 ♂ Cuatro Cienegas, 200 Ocampo St, Jul-1999, leg. A. Cohen, NVG-2284 | NVG140403-12 [TMMC]. **Mexico: Nuevo Leon:** 1 ♂ 28 km W Linares, 11-Apr-1979, leg. Schaffner & Friedlander, NVG-2240 | NVG140320-81 [TAMU]; 1 ♂ Hwy 60, ca 50 km WSW Linares, 2-Mar-1974, leg. R. O. Kendall & C. A. Kendall, NVG-2181 | NVG140320-22 [TAMU]; 1 ♂ ca 21 km WSW Cola de Caballo, 4 May 1978, leg. R. O. Kendall & C. A. Kendall, NVG-2182| NVG140320-23 [TAMU]. **Mexico: Tamaulipas:** 1 ♂ Gomez Farias, 500m, 19-Jul-1973, leg. Wm. McGuire, | KS019 [SDMC]; 1 ♀ Cd. Monte, Los Arcos Ct., 8-May-1978, leg. R. O. Kendall & C. A. Kendall, NVG-2185 | NVG140320-26 [TAMU]. **Mexico: San Luis Potosí:** 1 ♂ El Naranjo, 21-Feb-1976, leg. R. O. Kendall & C. A. Kendall, NVG-2183 | NVG140320-24 [TAMU]; 1 ♂ Hwy 70, 16 mi W Rioverde, 21-Feb-1980, R. O. Kendall & C. A. Kendall, NVG-2184 | NVG140320-25 [TAMU]. **Mexico: Morelos:** 1 ♂ Rancho Viejo, 20-Jul-2008, | KS034 [SDMC]. **Mexico: Veracruz:** 1 ♂ Fortín de las Flores, Motel Posada Loma, 900 m, 30-Mar-1977, leg. R. O. Kendall & C. A. Kendall, NVG-2186 | NVG140320-27 [TAMU]; 1 ♂ Fortin 13-Aug-1970, leg. B. Douglas, | KS038 [SDMC]. **Mexico: Oaxaca:** 1 ♂ Tlalixtac, 5 mi N of Oaxaca, 6000’, 13-IV-1988, leg. J. Kemner, NVG-2281 | NVG140403-09 [TMMC]; 1 ♂ Candelaria, Candelaria Loxicha, 1500’, 29-IV-1988, leg. J. Kemner, NVG-2282 | NVG140403-10 [TMMC]; 1 ♂ km 190 near Zanatepec, 500 ft, 24-Jul-1987, #14813, NVG-14102H01 [FMNH]; 1 ♂ Yangul, 1-Apr-1979, leg. J. Stoddard, | KS012 [SDMC]. **Mexico: Yucatan:** 1 ♂ Merida, Mar-1976, | KS021 [SDMC]. **Mexico: Quintana Roo:** 1 ♂ X-Can, 21-May-1963, | KS022 [SDMC]. **Honduras:** 1 ♀ Escuela Agrícola Panamericana, 30 km SE Tegucigalpa, 1-May-1985, leg. Vascones, NVG-2221 | NVG140320-62 [TAMU]; 1 ♂ 1 ♀ in copula, 18 km west of La Ceiba, 27-Jul-1980, leg. R. D. Lehman, NVG-14061A06, -14061A07 [USNM]. **El Salvador:** 1 ♂ San Salvador, 26-Sep-1971, leg. M. Serrano, NVG-14081G10 [CSUC]. **Costa Rica:** 1 ♂ Alajuela, Alajuela Adventist University of Centrali Americana, 15–20-May-1995, leg. J. C. Abbott & D. Petr, #308, NVG-2278 | NVG140403-06. **Panama:** 1 ♂ C. Z., La Pita, 19-Aug-1966, leg. G. B. Small, NVG-14061A05 [USNM].

##### Specimens excluded from the type series.

35 specimens from Texas (mostly central) possessed DNA barcodes of *Heraclides rumiko*, but many displayed morphological characters somewhat intermediate between those of *Heraclides rumiko* and *Heraclides cresphontes*. These specimens with full data are listed in Suppl. material 1 (as *Heraclides rumiko*, but not paratypes) and are excluded from the type series.

##### Type locality.

USA: Texas: Duval Co., Benavides, CR306, 1.8 mi west of SH339, latitude 27°36'27", longitude −98°26'29.4", elevation 124 m. This locality is at the sharp bend of the County Road 306, where several shrubs of Colima (*Zanthoxylum fagara* [L.] Sarg.) are growing by a fence. The egg that developed into the holotype was found on one of these shrubs.

##### Etymology.

The species is named to honor the wife of the first author. Pronounced as ’roo(as in rue)-mee(as in meek)-koh(as in cod). The stress is on the first syllable. The name is a noun in apposition.

##### Distribution.

*Heraclides rumiko* is recorded from the southwestern United States (mostly southern regions of four states: CA, AZ, NM, and TX) to Panama (DNA barcode data obtained for specimens from Mexico, El Salvador, Honduras, Costa Rica, and Panama). The northernmost barcoded specimen is from northeastern Colorado. In Mexico, *Heraclides rumiko* tends to be absent from deserts and high mountains, but is found elsewhere (Molina and León 2006). In central Texas, *Heraclides rumiko* is sympatric with its sister species *Heraclides cresphontes*. In Costa Rica and Panama, it is sympatric with *Heraclides homothoas* Rothschild & Jordan, 1906 (Tyler et al 1994), a likely sister to the ancestor of *Heraclides cresphontes* and *Heraclides rumiko*.

In the 1960s, *Heraclides rumiko* began expanding its distribution in California northward, and by the 1980s, it has reached central California (Emmel and Emmel 1973, Erickson and Iliff 2004). Citrus was cultivated in California from as early as 1840s (Laszlo 2007), and the factors that prevented *Heraclides rumiko* from northward expansion for 120 years are unknown. The increase in ornamental citrus trees may have supported the buildup of *Papilio rumiko* numbers in southern California and the butterfly can now be commonly found in urban areas. Rue, another host, is very commonly cultivated in southern California today.

##### Diagnosis.

**Figure 15.**
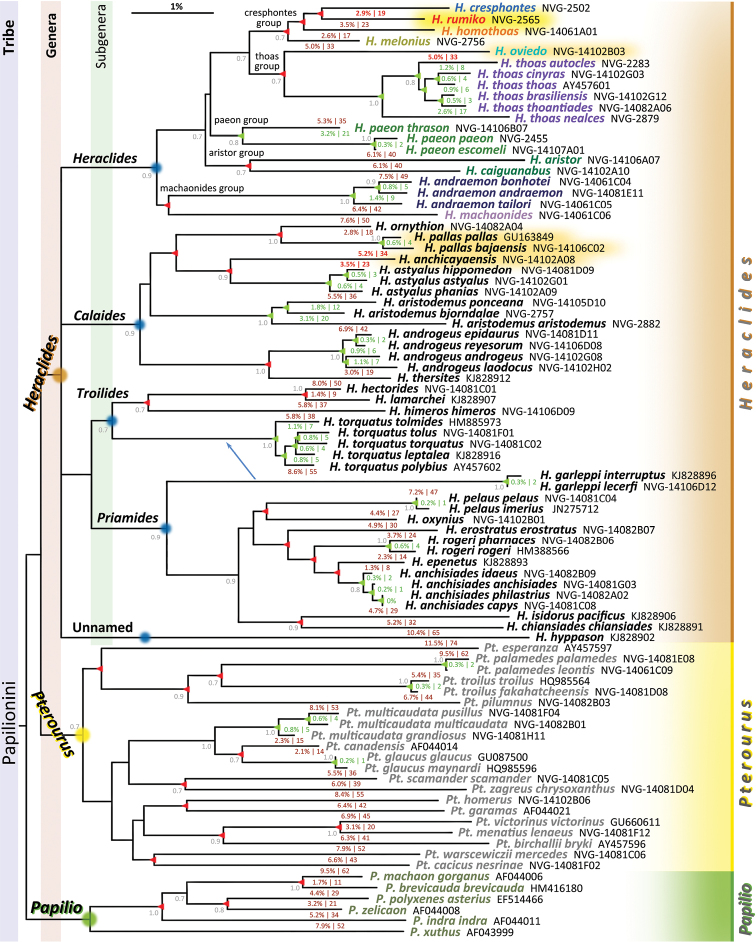
COI DNA barcode distances within *Papilionini* in a form of a BioNJ (Dereeper *et al*. 2008) dendrogram built using fraction of nucleotide differences between sequences as distance. The scale bar corresponding to about 1% difference in sequences is shown above the tree. Sequences obtained in this work are with “NVG-” number (see Supplementary Table 1 for data), others are from GenBank (http://genbank.gov/) and are labeled by accessions (letters and numbers, no dashes). Specimens with sequences from GenBank were not examined (except where a photograph was available from the BOLD database) and their identification follows original work, locality, and DNA barcode. Due to small number of phylogenetically informative positions, details of the tree topology especially closer to the root are not expected to be accurate (e.g., topology between subgenera of *Heraclides* remains unresolved and is shown as a quadfurcation) and the dendrogram is provided only to visualize the classification discussed in the text. Bootstrap values above 0.7 are shown by the nodes in gray font; “percent difference | number of differences” between the adjacent sequences in the dendrogram are shown between the branches. E.g., sequences of *Heraclides rumiko* and *Heraclides homothoas* differ by 3.5%, which is 23 base pairs. Differences between species are colored red (to substantiate new name, new status, and new combination) and maroon, and differences between subspecies within species are colored green. Nodes leading to speciation events are marked with red triangles and nodes leading to diversification into subspecies are marked with green triangles. Three Neotropical genera of Papilionini, five subgenera of *Heraclides* (one unnamed) and five proposed species groups in the subgenus *Heraclides* are labeled. New species described in this study is highlighted yellow and taxa with proposed changes in taxonomic status or name combination are highlighted orange. Arrow indicates that *Heraclides garleppi* belongs to subgenus *Troilides* by morphology, despite its COI barcode being more similar to *Priamides*.

**Figure 16.**
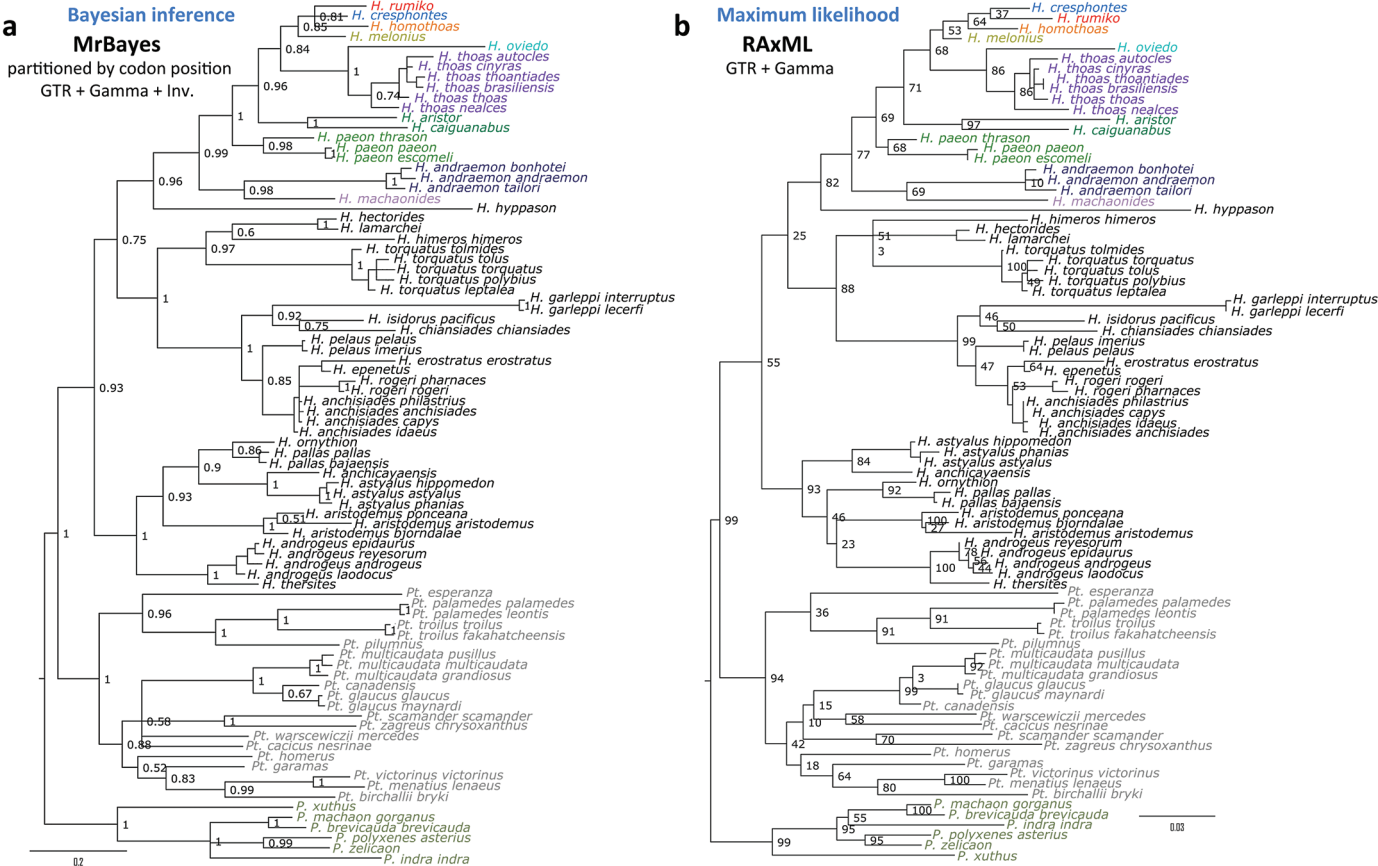
COI DNA barcode trees. Trees of representative sequences of Papilionini reconstructed with different methods: **a** Bayesian inference using MrBayes (alignment partitioned by codon position, nst=6, rates=invgamma, ratepr=variable), posterior probabilities are indicated by the nodes **b** Maximum likelihood method RAxML (-m GTRGAMMA), bootstrap values are indicated. Posterior probabilities are shown by the nodes (omitted within species). Names of different species are shown in different colors. Sequences obtained in this work are those with “NVG-” number (see Supplementary Table 1 for complete data), others are from GenBank (http://genbank.gov/) and are labeled by accessions (letters and numbers, no dashes). Specimens with sequences from GenBank were not examined (except where a photograph was available from the BOLD database) and their identification follows original work, locality, and DNA barcode.

**Figure 17.**
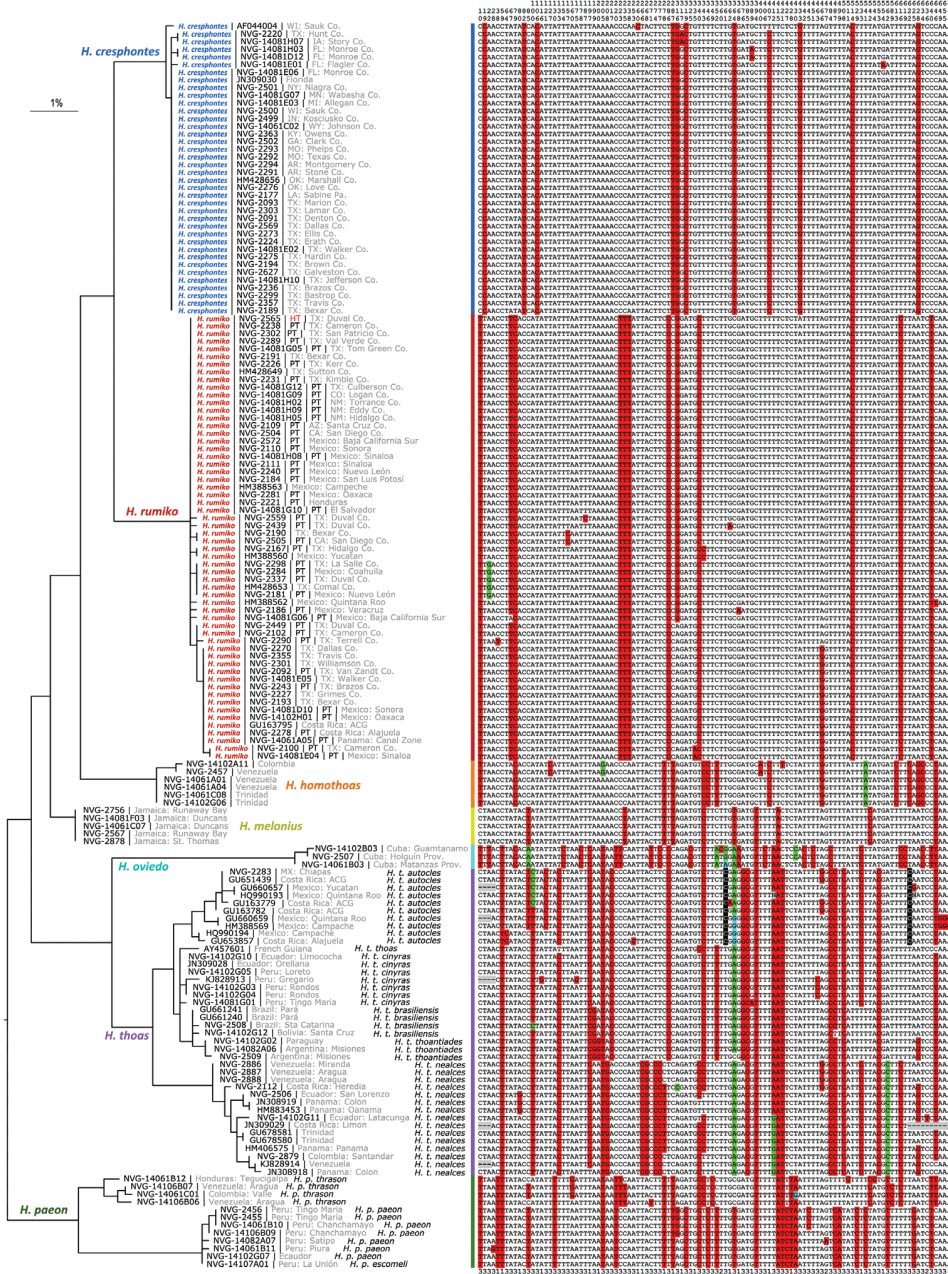
COI DNA-barcodes. Relationships between *Heraclides* specimens from the cresphontes, thoas and paeon groups in a form of RAxML (Stamatakis 2006) maximum likelihood tree (-m GTRGAMMA). Tree topology is the same as that of the distance tree (Fig. 15). The scale bar corresponding to about 1% difference in sequences is placed above the tree. Species names are shown by tree clades and are colored. Subspecies names are in black after localities. Sequences obtained in this work are labeled by “NVG-” number, those from GenBank (http://genbank.gov/) are labeled by accessions (letters and numbers, no dashes). Only selected sequences are shown (different haplotypes or different localities), data for additional NVG- specimens are in Supplementary Table 1. Barcode sequences with invariant positions removed are shown on the right. Positions are numbered according to the barcode sequence of *Heraclides rumiko* holotype (see text) and the numbers are shown above the sequences (e.g., the first position shown is 10 and the last is 655). Most common nucleotide in each position is not highlighted, less common are highlighted in color, giving each group of sequences “barcode”-like appearance different for each species. Location of each position in a codon (first, second, or third base) is shown below the alignment. Specimens with sequences obtained from GenBank were not examined (except where a photograph was available) and their identification follows DNA barcode.

**Figure 18.**
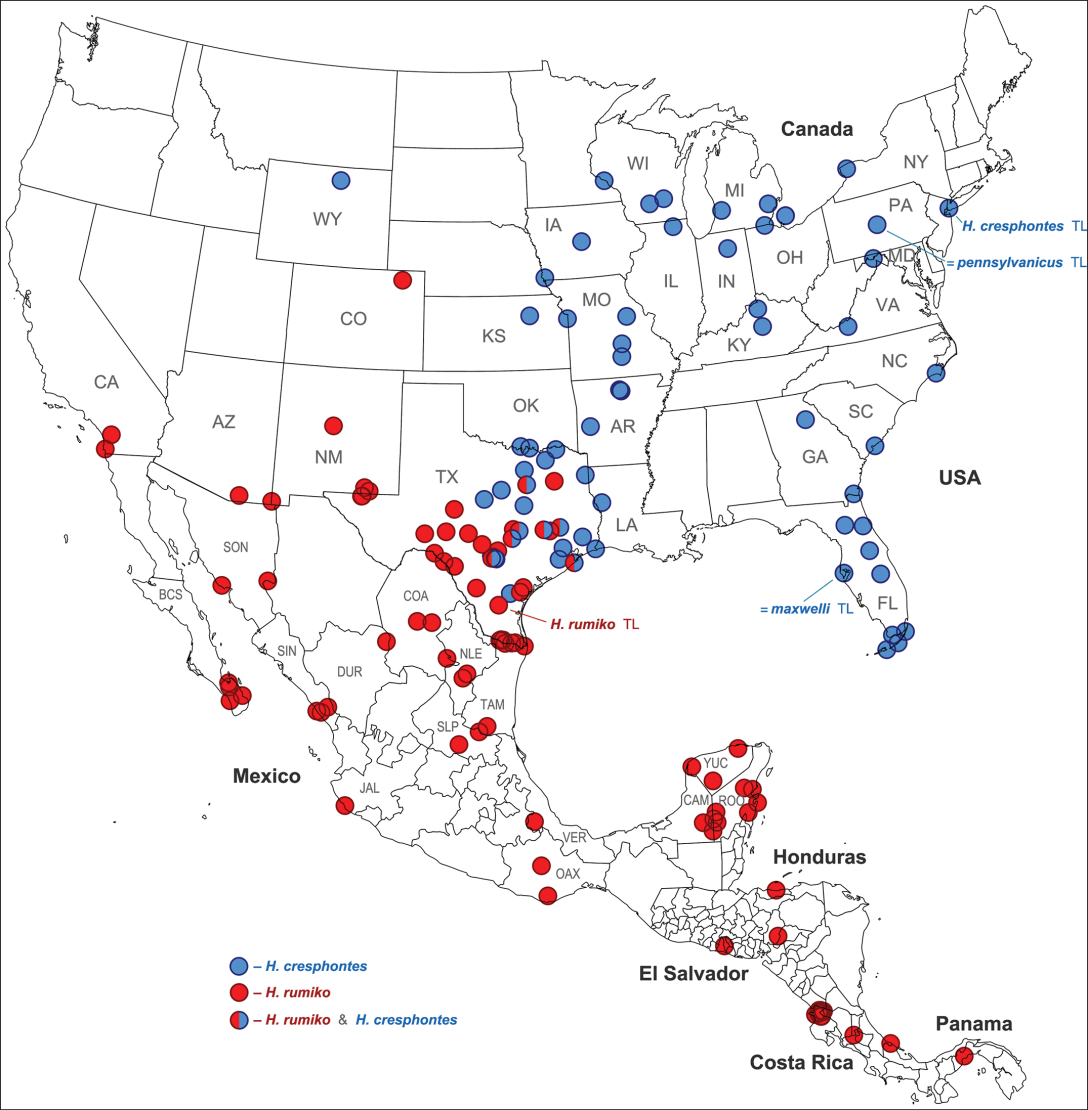
Localities of *Heraclides cresphontes* and *Heraclides rumiko* specimens with available DNA barcode information. Color of circles corresponds to species: *Heraclides cresphontes* - blue (based on 112 DNA COI barcode sequences, 103 obtained in this work); *Heraclides rumiko* - red (based on 183 barcodes, 146 obtained in this work), split red/blue circles mark localities where both *Heraclides cresphontes* and *Heraclides rumiko* were recorded. Type localities for taxa with available names are indicated with a corresponding name followed by “TL”. We treat Papilio cresphontes var. maxwelli Franck, 1919 & *Papilio cresphontes pennsylvanicus* F. Chermock & R. Chermock, 1945 as junior subjective synonyms of *Heraclides cresphontes*. Countries and states (for USA and Mexico) with records are labeled.

*Heraclides rumiko* belongs to the genus *Heraclides* Hübner, [1819] (type species *Heraclides thoas*), because it possesses simple, smooth, U-shaped juxta, which is a synapomorphy for the genus. *Heraclides rumiko* is in the subgenus *Heraclides* Hübner, [1819] (type species *Heraclides thoas*), because its uncus is shaped as two paired (i.e., uncus dorsad, brachium ventrad) horn-like processes, similarly to *Heraclides thoas*, and the harpe lacks a spine directed distad and separated at the end on its ventral surface. *Heraclides rumiko* is from the cresphontes group because the harpe is rounded, without sharp spines. These taxonomic attributions are also supported by the DNA barcode distances and trees (Fig. 15, 16). *Heraclides rumiko* differs from *Heraclides melonius* in lacking a knob-like blunt projection at the distal end of harpe and having restricted or absent orange scaling at the base of ventral hindwing cell M_1_-M_2_. *Heraclides rumiko* differs from *Heraclides homothoas* in having a simple beak-like, not bifid, pseuduncus. Compared to its sister species *Heraclides cresphontes*, *Heraclides rumiko* is characterized by (Figs 11, 13): (1) longer and more slender uncus arms, often strongly curving inwards; (2) brachium arms that project from the base of uncus on the inner (and not outer) side, and are visible below uncus in dorsal view, not hidden beneath uncus; (3) in lateral view, an uncus and brachium that point away from each other: posterodorsad (uncus) and posteroventrad (brachium), and not in the same direction; (4) bases of uncus and brachium that are weakly fused, with weaker sclerotization at the base of brachium; (5) two continuous yellow stripes dorsally from head to thorax, instead of separate spots; (6) more slender wings with longer tails; (7) smaller marginal yellow spots on the forewing; (8) a background-colored spot on yellow central band in cell R_5_-M_1_ that is larger, with better defined edges; (9) a submarginal forewing band mostly of 3 spots, not 4; (10) a black median band on ventral hindwing that is more expressed and straight; (11) a COI DNA barcode sequence that differs by about 3%. Seventeen positions are consistently invariant in either *Heraclides rumiko* or *Heraclides cresphontes*, but different between them on a sample of 183 *Heraclides rumiko* and 112 *Heraclides cresphontes* specimens across the range (Figs 17, 18, Suppl. material 1). These 17 positions are listed here in the format “k X (not Y)”, where k is a sequential number of the position (numbering is from 1 to 658 for the barcode sequence of *Heraclides rumiko* holotype shown above), X is a nucleotide in *Heraclides rumiko* barcodes and Y is a nucleotide in *Heraclides cresphontes* barcodes: 10 T (not C), 19 T (not C), 79 C (not T), 82 C (not T), 106 T (not C), 223 T (not C), 235 T (not C), 238 T (not A), 286 C (not T), 287 C (not T), 319 A (not C), 340 C (not T), 364 C (not T), 433 A (not G), 616 C (not T), 628 A (not G), 640 T (not C). While these positions distinguish the two species in a sample of 295 specimens, some of the positions may show variation when a larger sample of sequence is accumulated.

Female genitalia are very variable in both species (Fig. 12), and we were not able to find differences between the two species. Also, due to significant variation in many characters, not all specimens might be readily identifiable, especially in central Texas, where *Heraclides rumiko* and *Heraclides cresphontes* likely hybridize at least to some extent as judged from intermediate characters in some specimens (Fig. 11E). Male genitalia, stripes on the neck and DNA barcodes are the most reliable characters for identification. Genitalia morphometrics involving 3 measures completely separates the two species (excluding specimens from central Texas) with a hiatus between them when a weighted sum of the 3 measures is used (Fig. 11E, along horizontal axis). None of the three measures is sufficient when used separately due to variation. The best of the three, the angle between uncus and brachium in lateral view, identifies all specimens but one. The failed specimen possesses brachium strongly curved dorsad, but other two measures in its genitalia correctly identify this specimen. Linear combination of the measures is more robust to variation than a single measure and should be used for more confident identification. Similar results were obtained with a combination of 4 facies measures, one of which was the continuity of the stripe on the neck (Fig. 11E, along vertical axis).

##### Life history, foodplants, and phenology

**Figure 19.**
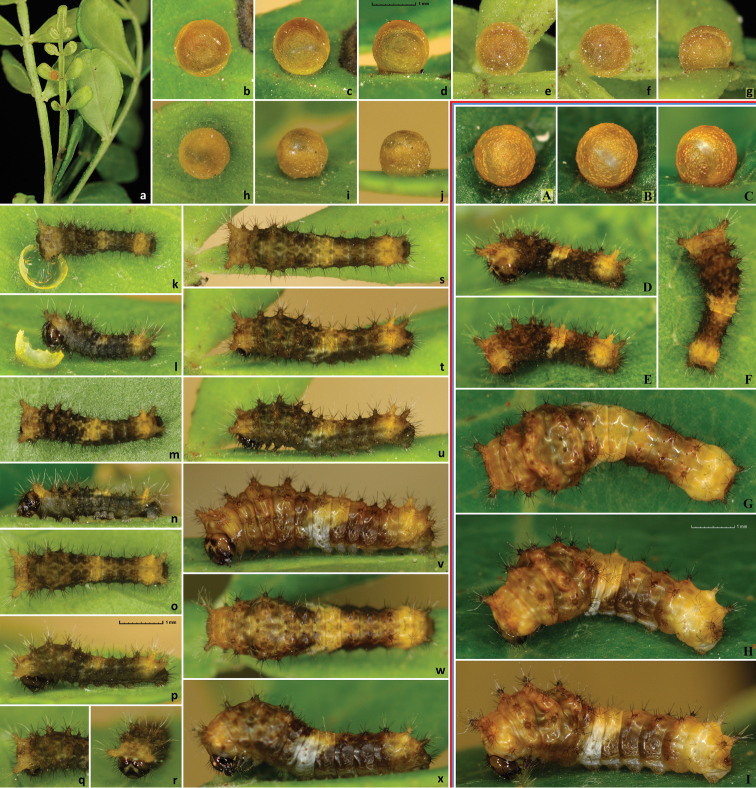
Life history: eggs and 1^st^ instar caterpillars. **a–x** *Heraclides rumiko* types, USA: TX: Duval Co., Benavides **A–I** *Heraclides cresphontes*, USA: TX: Denton Co., Grapevine Lake, Murrell Park **a–j**, **A–C** ova; **k–x**, **D–I** 1^st^ instar caterpillars; 1 mm scale shown on panels **d**, **p** and **H** refers to all images except **a**, which shows a typical position for an egg on a fresh leaf. In Figs 19–23, dorsal, dorsolateral, and lateral views are shown for most individuals, supplemented with anterior and posterior views for some caterpillars. Sexes and voucher numbers (where available) and dates: **a**, **e**–**g** paratype ♀ NVG-2564, 19-Apr-2014 **b–d** holotype ♂ NVG-2565, 19-Apr-2014; **h**–**l** paratype ♀ NVG-2563, 19-Apr-2014 (**h–j**), ♀ NVG-2563, 20-Apr-2014 (**k–l**) **m–n** paratype ♂ NVG-2559, 19-Apr-2014 **o–r** paratype ♀ NVG-2564, 22-Apr-2014 **s–u** paratype ♂ NVG-2559, 20-Apr-2014 **v** holotype ♂ NVG-2565, 25-Apr-2014 **w–x** paratype ♂ NVG-2559, 22-Apr-2014 **A–I** ♂ NVG-2760,16-Jun-2014 (**A–C**), 19-Jun-2014 (**D–F**), 21-Jun-2014 (**G–I**).

**Figure 20.**
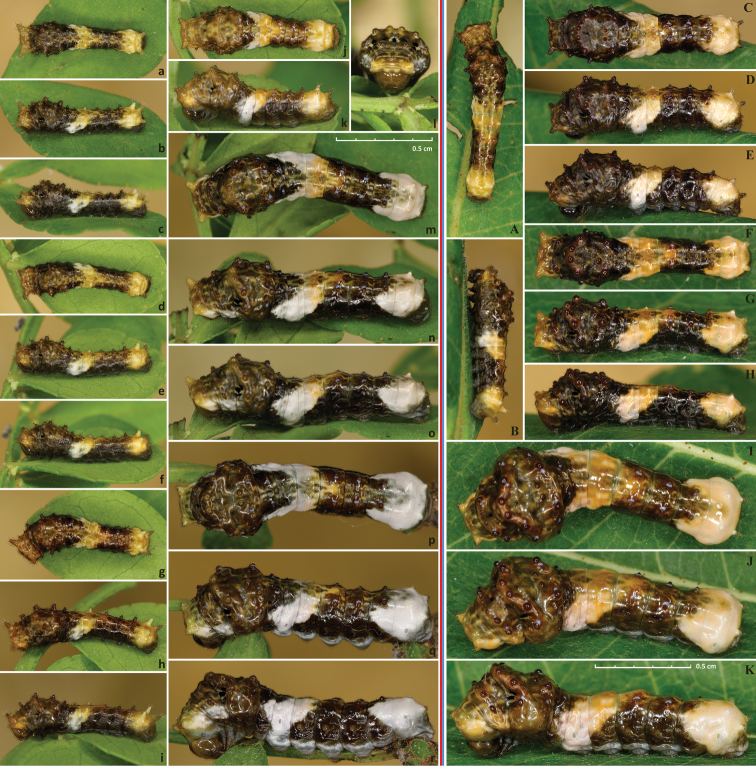
Life history: 2^nd^ and 3^rd^ instar caterpillars. **a–r** *Heraclides rumiko*, USA: TX: Duval Co., Benavides **A–K** *Heraclides cresphontes*, USA: TX: Denton Co., Grapevine Lake, Murrell Park **a–k**, **A–E** 2^nd^ and **k–r**, **F–K** 3^rd^ instar caterpillars; 0.5 cm scale shown on panels **m** and **K** refers to all images. Sexes and voucher numbers (where available) and dates: **a–c** paratype ♀ NVG-2564, 25-Apr-2014 **d–f** paratype ♀ NVG-2563, 25-Apr-2014 **g–i** larva #7 (died), 29-Apr-2014 **j–k** paratype ♂ NVG-2559, 25-Apr-2014 **l–o** paratype ♂ NVG-2559, 27-Apr-2014 **p–r** paratype ♀ NVG-2564, 29-Apr-2014 **A–B**, **F–K** ♂ NVG-2760, 22-Jun-2014 (**A–B**), 24-Jun-2014 (**F–H**), 26-Jun-2014 (**I–K**) **C–E** ♀ NVG-2741, 16-Jun-2014.

**Figure 21.**
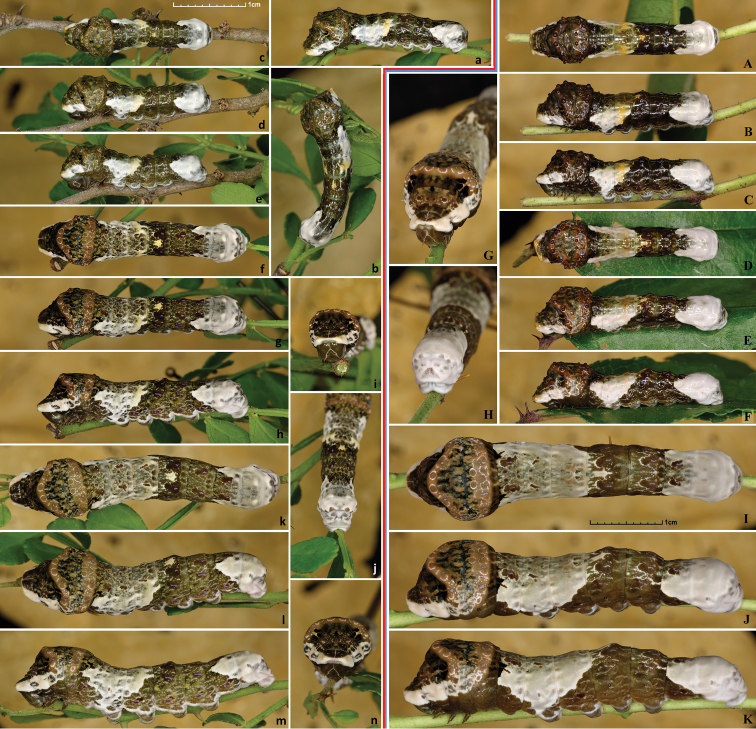
Life history: 4^th^ and 5^th^ instar caterpillars. **a–n** *Heraclides rumiko*, USA: TX: Duval Co., Benavides **A–K** *Heraclides cresphontes*, USA: TX: Denton Co., Grapevine Lake, Murrell Park **a–e**, **A–F** 4^th^ and **f–n**, **G–K** 5^th^ instar caterpillars; 1 cm scale shown on panels **c** and **I** refers to all images. Sexes and voucher numbers (where available) and dates: **a–b**, **f–n** is the same individual (larva #2, died, shown in Fig. 22a–c), 24-Apr-2014 (**a–b**), 29-Apr-2014 (**f–j**), 29-Apr-2014 (**k–n**) **c–e** larva #1 (died), 24-Apr-2014 **A–C** ♂ NVG-2740, 16-Jun-2014 **D–F** ♀ NVG-2741, 21-Jun-2014 **G–K** ♀ NVG-2741, 26-Jun-2014.

**Figure 22.**
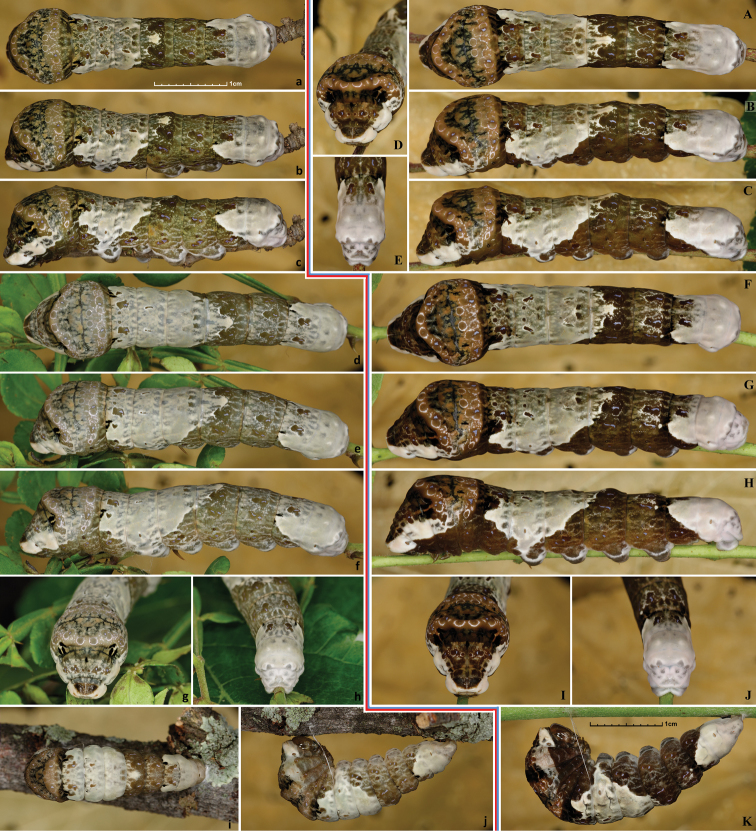
Life history: 5^th^ instar caterpillars and prepupae. **a–j** *Heraclides rumiko*, USA: TX: Duval Co., Benavides **A–K** *Heraclides cresphontes*, USA: TX: Denton Co., Grapevine Lake, Murrell Park **a–h**, **A–J** 5^th^ instar caterpillars and **i–j**, **K** prepupae; 1 cm scale shown on panels **a** and **K** refers to all images. Sexes and voucher numbers (where available) and dates: **a–c** larva #2, died (shown in Fig. 21a–b, f–n), 1-May-2013 **d–h** paratype ♀ NVG-2564, 11-May-2014 (adult Figs 9, 10) **i–j** paratype ♂ NVG-2559, 11-May-2014 **A–E** 26-Jun-2014 **F–K** ♂ NVG-2740, 21-Jun-2014 (**F–J**) & 23-Jun-2014 (**K**).

**Figure 23.**
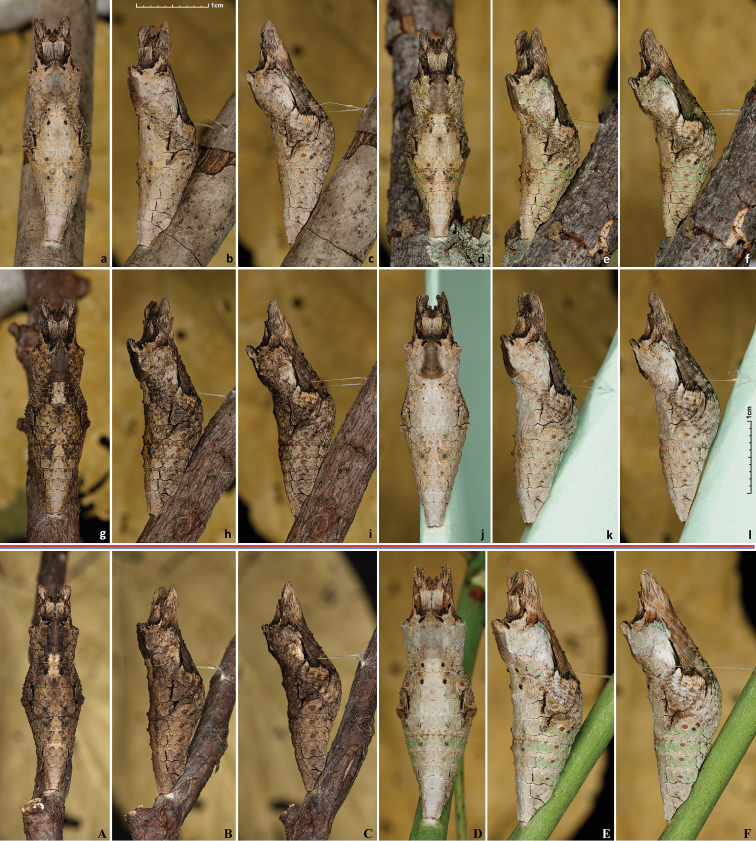
Life history: pupae. **a–l** *Heraclides rumiko* types, USA: TX: Duval Co., Benavides **A–F** *Heraclides cresphontes*, USA: TX: Denton Co., Grapevine Lake, Murrell Park; 1 cm scale shown on panels **b** and **l** refers to all images. Sexes and voucher numbers (where available) and dates: **a–c** holotype ♂ NVG-2565, 17-May-2014 **d–f** paratype ♂ NVG-2559, 17-May-2014 **g–i** paratype ♀ NVG-2563, 17-May-2014 **j–l** paratype ♀ NVG-2564, 17-May-2014 (adult Figs 9, 10) **A–F** 28-Sep-2014 **D–F** ♂ NVG-2740, 26-Jun-2014.

**Figure 24.**
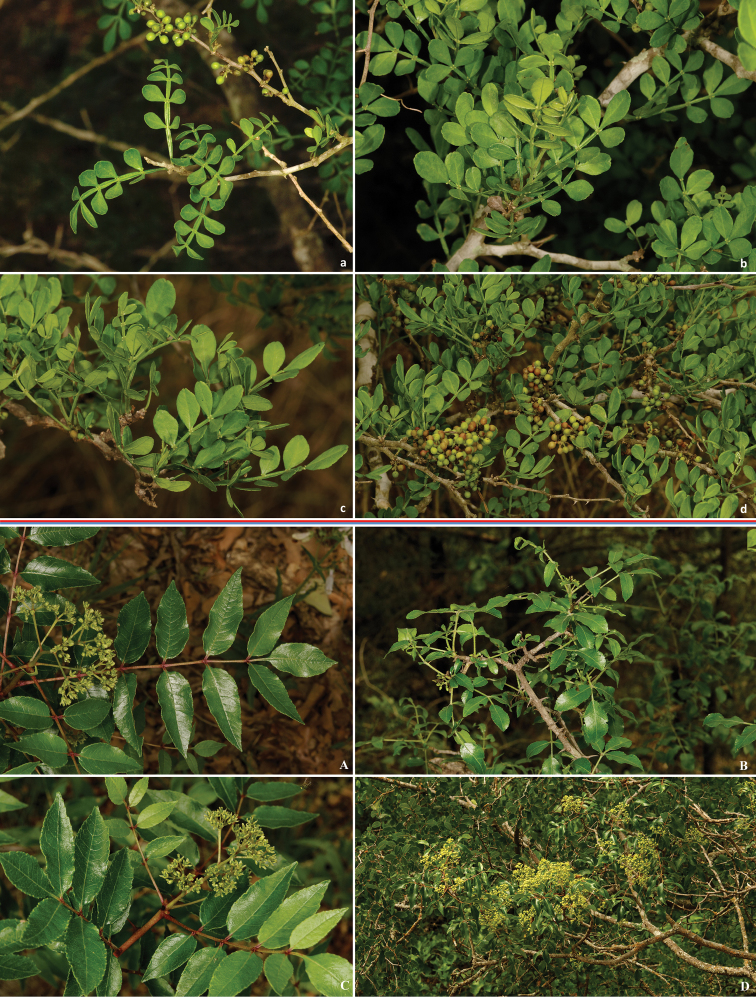
Foodplants most commonly used by *Heraclides* caterpillars. **a–c** *Zanthoxylum fagara* [L.] Sarg (Colima, Lime Prickly-ash), USA: TX: Duval Co., Benavides, 19-Apr-2014, used by *Heraclides rumiko* **A–C** *Zanthoxylum clava-herculis* L. (Hercules’s club, Pepperwood), USA: TX: Denton Co., Grapevine Lake, Murrell Park, 26-Apr-2014, used by *Heraclides cresphontes*.

(Figs 19–24). The following observations were made in Texas and southern California. Eggs are laid singly on young leaves (Fig. 19a) and shoots of the host plants from Rutaceae family: *Zanthoxylum fagara* [L.] Sarg, *Ptelea trifoliata* L., *Amyris texana* (Buckley) P. Wilson, and *Casimiroa greggii* (S. Watson) F. Chiang in Texas, or *Ruta graveolens* L. and *Citrus* spp. in both states, and likely others. For instance, ovipositions were recorded on *Geijera parviflora* Lindl. in Tucson, Pima Co., Arizona and females were observed around these trees in Los Angeles County, CA (Brian Banker, personal observations). Eggs are round, 1.1–1.6 mm in diameter, coated with a substance giving the surface a granular appearance (Fig. 19b–j). Color of egg is pale yellow when laid, gradually changing to dull orange-brown. *Heraclides rumiko* eggs are typically smaller than *Heraclides cresphontes* eggs and are finer grained on the surface (Fig. 19A–C). Eggs hatch in 7–10 days. Prior to hatching, larval head can be seen through the eggshell.

Larva eats egg shell upon hatching (Fig. 19k, l). 1^st^ instar is 3–5 mm in length (Fig. 19k–x), covered with prominent setae, head capsule yellow-brown with dark-brown spots and paler caret in the middle, body pattern resembles bird-droppings similarly to many other Papilionidae. Larva bears this pattern through the end of development, but it charges somewhat between instars. 2^nd^ instar is 5–11 mm (Fig. 20a–l), head from this instar on is uniformly brownish without darker spots. 3^rd^ instar is 11–16mm (Fig. 20k–r), 4^th^ instar is 16–30.0 mm (Fig. 21a–e), and 5^th^ instar is 30–50 mm (Figs 21f–n, 22a–h). While the first 4 instars appear shiny, the 5^th^ one is matte. Larval growth is rapid under indoor rearing condition with ambient room temperature, taking only about 9 days to reach ultimate instar. When a late instar larva is startled, it lifts its head and inflates the thorax, revealing the eyespots on meta-thorax (Fig. 22g). If disturbed further, it everts red osmeterium from behind the head. Early instar larva tends to use osmeterium right away when disturbed, and osmeterium of the first instar is yellowish. Right before pupation, the color of larva changes little, still resembling bird droppings (Fig. 22i, j), but becoming slightly more uniform and paler. Much more drastic prepupal color changes are observed in some other Papilionidae, such as *Pterourus rutulus* (Lucas, 1852) and *Pterourus eurymendon* (Lucas, 1852), whose larvae turn from green to brown (Shiraiwa 2009). Since the larva is searching for suitable pupation sites on brown tree branches rather than green leaves, such color change is adaptive. Ultimate instar *Heraclides* larvae are already brown and rest on branches and not leaves to begin with. *Heraclides cresphontes* caterpillars (Figs 19D–I, 20A–K, 21A–K, 22A–K) are very similar to *Heraclides rumiko*, but most caterpillars are browner, more vividly colored and are less gray and less green, especially in the ultimate instar.

Pupa, 26–36mm in length (Fig. 23a–l), is mottled pale to grayish and dark brown, resembling surface of a tree or branch it is attached to. Some pupae develop greenish-olive spots (Fig. 23d–f). The darkness of a pupa is frequently determined by the color of the surface it rests on. E.g., the darkest pupa was formed on a dark branch (Fig. 23g–i). The palest one (Fig. 23j–l) is attached to a pale-green paper sheet covering the pupation container with the goal to induce a pale pupa. The pupa on a branch with greenish lichens developed the largest amount of green (Fig. 23d–e). *Heraclides cresphontes* pupae are very similar, but are typically larger (Fig. 23D–F), although poor foodplant condition could result in smaller pupae (Fig. 23A–C). Adult either emerges from pupa in 1 to 2 weeks, or pupa goes into diapause for several months. Pupae overwinter. Pupae can be brought out of diapause prematurely with increase of temperature (Brian Banker, unpublished). Due to dark coloration of pupal wing cases, it is difficult to see when the adult is ready to eclose. The best indicator of pupae being near eclosion might be the dark stripe showing through the median on the dorsal side of an abdomen, which are usually paler and most transparent in pupae.

In south Texas (e.g., near the type locality in Duval County), *Heraclides rumiko* larvae are found on small to medium size Colima shrubs, *Zanthoxylum fagara* [L.] Sarg (Fig. 24a–d) throughout ranches and particularly along the roads, which males use as flying corridors. Early instar larvae rest on leaves, ultimate instar caterpillars rest on branches, which they resemble in color. Adults can be found on wing during most of the year except the coldest months. Adults are more common from April to September, when emergences peak every 1.5 months with exact timing dictated by the severity of winter and the amount of rain. In southern California adults fly from late February through mid-November. In San Diego, California, the larvae are often found on citrus trees in gardens and orchards. The adults frequent the cities where many ornamental citrus plants grow, and the number of adults seen increases during August and September. *Heraclides cresphontes* in Texas uses Pepperwood *Zanthoxylum clava-herculis* L. (Fig. 24A–D) as the major caterpillar host.

**Figure 25.**
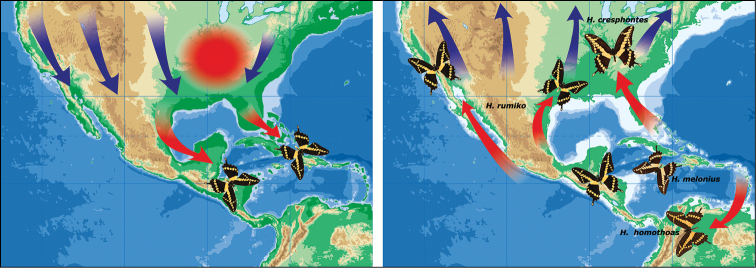
Speculations about origins of the *Heraclides cresphontes* group species. Left panel: Ancestor of *Heraclides cresphontes* might have originated in eastern North America (red area), speciating from a more southern *Heraclides thoas*. During Pleistocene, glaciation and cooler climate (blue arrows) forced *Heraclides cresphontes* ancestor south through two possible paths: to Mexico and to Florida-Caribbean islands (red arrows). Resulting populations got isolated and differentiated into four species. Right panel: With retreat of glaciation (blue arrows), eastern *Heraclides cresphontes* moved back to eastern North America; southwestern *Heraclides rumiko* moved north through west and east Mexico; *Heraclides melonius* got trapped and speciated further in Jamaica. *Heraclides homothoas* probably evolved in northern South America arriving from the Caribbean islands through Trinidad and moved north to Costa Rica thus overlapping with *Heraclides rumiko* in distribution.

## Taxonomic adjustments in *Heraclides*

**Subgenera of *Heraclides*.** To gain better understanding of *Heraclides* taxonomy, we determined new and retrieved available COI DNA barcodes for a number of species and subspecies from the Tribe Papilionini. The resulting distance dendrogram and trees are shown in Figs 15, 16. Although our results are not expected to fully reflect phylogeny accurately due to short length of DNA barcodes (654 base pairs) and possible discordance between mitochondrial and nuclear genomes, it agrees very well with the results obtained on a much larger sample of positions (Zakharov et al. 2004)—i.e., the three genera of Neotropical Papilionini: *Papilio* Linnaeus, 1758, *Pterourus* Scopoli, 1777, and *Heraclides* Hübner, [1819] are monophyletic and well-separated in all analyses and will all substitution models. Topology within each genus also mostly agrees with that obtained previously (Zakharov et al. 2004), although position of certain branches like *Pterourus esperanza* (Beutelspacher, 1975) and *Pterourus homerus* (Fabricius, 1793) might have been influenced by long branch attraction in some trees (Figs 15, 16). The differences in topology between the trees obtained by different methods are mostly confined to less confident nodes as judged by Bootstrap, Posterior probability and Bremer support values, and typically involve the order of basal branching in each genus or near the leaves of the tree (between subspecies). It should be noted that to simplify illustrations, each taxon is represented by a single sequence on Figs 15 and 16a–b. However, for most taxa we obtained (Suppl. material 1) and analyzed several sequences frequently from different localities.

Species in the genus *Heraclides* group into five prominent clades that can be treated as subgenera (Fig. 15). Four of these subgenera have been named: the nominate (type species: *Heraclides thoas*); *Calaides* Hübner, [1819] (type species: *Heraclides androgeus* (Cramer, 1775)); *Troilides* Hübner, [1825] (type species: *Troilides tros* Hübner, [1825], considered a subjective synonym of *Heraclides torquatus polybius* (Swainson, 1823)); *Priamides* Hübner, [1819] (type species: *Priamides hipponous* Hübner, [1819], considered a subjective synonym of *Heraclides anchisiades anchisiades* (Esper, 1788)). The fifth subgenus is unnamed and consists of a single species, *Heraclides hyppason* (Cramer, 1775), which is characterized by unique phenotype, thus it is not surprising that it stands out from the rest. Statistical support for the relationships between the five subgenera is low, and their branching order is inconsistent between different methods, except that *Troilides* is a likely sister of *Priamides*. Therefore we show the rest as a quadfurcation in the dendrogram (Fig. 15), assuming the order between them unresolved. Interestingly, in many analyses, *Heraclides hyppason* was placed as a sister to *Heraclides* (Fig. 16a) despite significant differences in facies. This placement of *Heraclides hyppason* was also suggested by Lewis et al. (2014). Regardless of its exact placement, both studies suggest significant divergence of *Heraclides hyppason* from other taxa. also apparent from its facies.

**Subgenus *Heraclides*.** Species with available barcode sequences in the subgenus *Heraclides* can be partitioned into five species groups. The cresphontes group includes four species: *Heraclides cresphontes*, *Heraclides rumiko*, *Heraclides homothoas*, and *Heraclides melonius*. Relationships between them are statistically unresolved, but the tree topology is reasonable. *Heraclides melonius* splits out first, in agreement with its isolation in Jamaica and more significant differences in genitalia of both sexes: e.g., long pseuduncus (*Heraclides melonius* was originally described as a subspecies of *Heraclides thoas*), harpe with a terminal knob, vestigial labella postvaginalis, and much enlarged vestibular figs. *Heraclides rumiko* is a sister to *Heraclides cresphontes* in accord with pronounced similarities between these two species.

Analysis of the thoas group revealed that the Cuban taxon is very distant from the rest, showing more than 5% difference in COI barcodes, a difference much larger than the divergence within the cresphontes group falling within 3.5% (Figs 15, 16). This barcode difference correlates well with pronounced genitalic differences, both in males and females: valva is more elongated and with a longer harpe extending into a terminal spine almost reaching the distal end of valva; lamella postvaginalis is smaller and vestibular figs are more robust with larger, and almost square, inner lateral processes. Its wings are characterized by deeper yellow ventral side with prominent blue patches on hindwing. These differences imply that *Heraclides oviedo* (Gundlach, 1866), **reinstated status**, is a strongly differentiated species and not a subspecies of *Heraclides thoas*.

In agreement with Lewis et al. (2014), endemics of Hispaniola and Cuba *Heraclides aristor* and *Heraclides caiguanabus* fall within the subgenus *Heraclides*, and we define the aristor group to encompass these two sister species (Fig. 15). The exact placement of this clade within the subgenus was inconsistent between different methods and parameter sets, but it is possible that they are closer related to cresphontes and thoas groups than the paeon group (Fig. 16). Indeed, morphologically, these Caribbean species are similar to *Heraclides cresphontes* and *Heraclides thoas* (e.g., more robust uncus is more similar to *Heraclides thoas* than to *Heraclides paeon*), but appear visually distinct due to the absence of the central band across wings.

The machaonides group consists of two species: *Heraclides machaonides* (Esper, 1796) and very distant from it *Heraclides andraemon* Hübner, [1823] with its three subspecies: nominate, *Heraclides andraemon bonhotei* (Sharpe, 1900), and *Heraclides andraemon tailori* (Rothschild & Jordan, 1906), which are rather close to each other in DNA barcodes (within 1.5%). Limited divergence in DNA is consistent with morphological similarities, and these three taxa are best treated as subspecies of *Heraclides andraemon*.

**Subgenus *Calaides*.** While we have not performed detailed analysis of other subgenera in *Heraclides*, we notice and correct two inconsistencies between the current taxonomy and similarities of DNA barcodes in the subgenus *Calaides* (Figs 15, 16). In agreement with Lewis et al. (2014), we see that *Heraclides astyalus* (Godart, 1819) is paraphyletic with regard to *Heraclides ornythion* (Boisduval, 1836) (Figs 15, 16). Thus, *Heraclides pallas* (G. Gray, [1853]), **reinstated status**, with its subspecies *Heraclides Papilio bajaensis* (J. Brown & Faulkner, 1992), **new combination**, are not conspecific with *Heraclides astyalus* (Godart, 1819). Indeed, DNA barcode difference between *Heraclides pallas* and *Heraclides astyalus* is 4.6%, which is significantly larger than the 2.6% between *Heraclides pallas* and *Heraclides ornythion*. In male genitalia, *Heraclides pallas* differs from *Heraclides astyalus* in having a thicker and shorter spike on the ventral side of harpe (the spike does not extend much past the harpe distal end), and the rasp-like ridge of harpe is perpendicular to the smooth edge of harpe (Lewis 2010, Rothschild and Jordan 1906). In *Heraclides astyalus*, the spike protrudes distad clearly beyond the end of harpe and almost reaches the edge of valva, and the rasp-like ridge of harpe is almost parallel to the smooth edge of harpe (Lewis 2010, Rothschild and Jordan 1906). In female genitalia of *Heraclides pallas*, the inner lateral processes of the vestibular figs are wider, with a larger number of smaller teeth at the edges (Lewis 2010). Furthermore, we see (Figs 15, 16) that *Heraclides anchicayaensis* Constantino, Le Crom & Salazar, 2002, **new status**, described from western Colombia and characterized by narrower bands and dorsal hindwing submarginal lunules differs by 3.5% in barcode from *Heraclides astyalus* populations, which have very similar barcodes from Colombia to Argentina in all three subspecies. Additionally, *Heraclides anchicayaensis* is apparently sympatric with *Heraclides astyalus hippomedon* (C. Felder & R. Felder, 1859) in Colombia (Lewis 2010).

**Discrepancy between barcodes and morphology.** Generally, we see excellent agreement of the DNA barcode trees (Figs 15, 16) with phylogeny obtained on longer sequences (Zakharov et al. 2004) and traditional views about relationships between Papilionini (Tyler et al. 1994). However, the most obvious discrepancy is the placement of *Heraclides garleppi* (Staudinger, 1892) within *Priamides* by DNA barcodes, while wing patterns (and traditional view based on similarities to *Heraclides torquatus* (Cramer, 1777)) argue for its affinity with *Troilides* (Lewis 2010, Tyler et al. 1994). It has been suggested that *Heraclides garleppi* might be a hybrid (Tyler et al. 1994), or a taxon of hybrid origin. Distinctness of genitalia and DNA barcodes argues that it is a good species, but the presence of a barcode very different from *Heraclides torquatus* may indeed suggest hybridizations leading to the origin of this species. This discrepancy between barcodes and morphology needs to be investigated, but Lewis et al. (2014) show the same position of *Heraclides garleppi* in their tree as in ours, and their results use morphological characters in addition to DNA barcodes. Regardless of such discrepancies, the major conclusions of this section, while hinted by DNA barcodes, are substantiated by characters in male and female genitalia and facies, and are consistent with the knowledge about Papilionini from the literature (Tyler et al. 1994).

### Synonymic list for the *Heraclides cresphontes* group

To summarize the nomenclature of the H. cresphontes group, we provide a synonymic list of its species. Name combination from the original description is used for each synonym (= subjective synonyms; =† objective synonyms; =‡ unavailable names) and for species is given after “|”. Format of the data: reference to the description | category of a primary type (**HT** holotype, **ST** syntypes, **LT** lectotype, **NT** neotype) - type locality; collection where primary types are stored. Inferred information is placed in brackets []. Type locality is given as a geographic position, not verbatim from the original description.

Genus ***Heraclides*** Hübner, [1819]

Verz. bekannt. Schmett. (2): 83-84. Type species: *Papilio thoas* Linnaeus, 1771; designated by Scudder (1875) Proc. Am. Acad. Arts Sci., Boston 10(2): 187, no. 517

Subgenus ***Heraclides*** Hübner, [1819]

=†*Thoas* Swainson, 1833

Zool. Illustr. (2)3(26): pl. 121, unnumbered text. Type species: *Papilio thoas* Linnaeus, 1771; by tautonymy

cresphontes species group

***Heraclides cresphontes*** Cramer, [1777] | *Pap*[*ilio*]. *Equ*[*es*]. *Achiv*[*us*]. *Cresphontes* | Eastern Giant Swallowtail

Uitl. Kapellen 2(14): 106-107, pl. 165 f. A ♀ D&V, pl. 166 f. B ♂ D (LT) | **NT** - USA: NY: Brooklyn; USNM

=†*Heraclides Oxilus* Hübner, [1819]

Verz. bek. Schmett. (2): 83 (replacement name for *Heraclides cresphontes*)

=‡*Papilio cresphontes* ab. (nov.) *lurida* Schultz, 1908

Entomol. Z. 22(23): 92 | **ST** - “North America”; ?; assignment to *Heraclides cresphontes* is speculative

=‡Papilio thoas cresphontes ab. luxuriosa Reiff, 1911

Z. wiss. InsektBiol. 7(5/6): 159 | **HT** - USA: MI: Detroit; MCZ

=‡Papilio cresphontes ab. intacta Strand, 1918

Soc. Ent. 33(12): 47; referred to Seitz (1907) Gross-Schmett. Erde 5: pl. 7 f. a [2] | **ST** - ?; ?

= Papilio cresphontes var. maxwelli Franck, 1919

Bull. Brooklyn Ent. Soc. 14(1): 3, f. 2 ♂ D (HT) | **HT** - USA: FL: Pinellas Co., St. Petersburg; USNM

=‡Papilio cresphontes tr. f. forsythae Gunder, 1933

Can. Ent. 65(8): 171 | **HT** - USA: FL: Miami-Dade Co., Florida City; AMNH

= *Papilio cresphontes pennsylvanicus* Chermock & Chermock, 1945

Proc. Penn. Acad. Sci. 19: 38-39 | **HT** - USA: PA: Centre Co., State College; CMNH

***Heraclides rumiko*** Shiraiwa & Grishin, 2014 | *Heraclides rumiko* | Western Giant Swallowtail

ZooKeys 468: 85–135 | **HT** - USA: TX: Duval Co., Benavides; USNM

=‡Papilio cresphontes forma melanurus Hoffmann, 1940

An. Inst. Biol. Univ. Méx. 11(2): 633-634 | **ST** - Mexico, Guerrero; AMNH

***Heraclides homothoas*** (Rothschild & Jordan, 1906) | *Papilio homothoas* | False Giant Swallowtail

Novit. Zool. 13(3): 561-562, no. 67 | **ST** - Venezuela: Ciudad Bolivar, Lower Orinoco; BMNH

***Heraclides melonius*** (Rothschild & Jordan, 1906) | *Papilio thoas melonius* | Jamaican Giant Swallowtail

Novit. Zool. 13(3): 556, no. 66a | **HT** - Jamaica; BMNH

## Discussion

The Giant Swallowtail *Heraclides cresphontes* is one of the largest butterflies in the United States, found mainly in the eastern US, from southern Canada to Florida and central Texas (Figs 14, 18). In this study, we assembled evidence that the North American southwest, from California to central Texas and south to Panama, is inhabited by its sister cryptic species (Bickford et al. 2007) that we named *Heraclides rumiko*. It is rather unusual for such a large butterfly species to remain unnamed, at least as a subspecies, but its superficial similarity to *Heraclides cresphontes* is a likely explanation. The two species differ from each other very subtly. They are mostly allopatric, and their ranges overlap in central Texas, around San Antonio and Austin, where both species are equally common and most likely hybridize (Fig. 11E). It is unclear why *Heraclides cresphontes* does not disperse south and *Heraclides rumiko* does not invade north, because they are excellent fliers and are likely to produce viable hybrids like many other Papilionidae do. In this section, we raise more speculative points about taxonomy, speciation, and evolution.

### *Heraclides* vs. *Papilio* s. l.

We follow Tyler et al. (1994) and Lamas (2004) in dividing Neotropical Papilionini into three genera: *Papilio*, *Pterourus*, and *Heraclides*. All recent studies show that these genera are monophyletic, can be defined by synapomorphies, and include sufficient number of species in each genus to be meaningful (Caterino and Sperling 1999, Zakharov et al. 2004, Simonsen et al. 2011). In some recent works, *Papilio* sensu lato is used as a genus that absorbs these three genera (Simonsen et al. 2011, Lewis at el. 2014). However, an unusual situation emerges when a subgenus name (i.e., *Heraclides*) is referred to more frequently in such works, and is therefore more instructive than the genus name. The level at which phylogenetic hierarchy is cut through to define genera is arbitrary and is for convenience of communication. Therefore, genera can be chosen as the most informative and major clades of species below the family and tribe levels. We think that for the New World representatives of the tribe Papilionini, *Papilio*, *Pterourus*, and *Heraclides* offer the most informative groupings, both from morphological and molecular standpoints.

First, divergence within each of the three genera is already very significant, reaching 10% sequence difference in the COI DNA barcode. In recent work on Lycaenidae, new criteria were proposed to delineate genera, i.e., “genera can be recognized as those lineages that originated in the late Miocene (older than 5 Myr)” (Talavera et al. 2012). Many of such genera differ by 6% and less in the barcodes. While we are not suggesting the use of a stringent universal time or DNA sequence difference threshold for genera identification, some consistency in genetic divergence across butterfly groups seems appealing. The age of *Papilio* s. l. has been estimated at over 50 Myr (Zakharov et al. 2004). Even the age of *Heraclides* was suggested to exceed 20 Myr (Lewis et al. 2014). To put these estimates in perspective, divergence between *Homo* Linnaeus, 1758 and *Pan* Oken, 1816—the genus closest to *Homo*—is estimated to be about 10 Myr (Arnason et al. 1998) with COI barcode difference being about 10%.

Second, each of the three Papilionini genera can be further divided into meaningful subgenera to denote finer groupings of species that correlate with phylogeny and morphology. For instance, in *Heraclides*, our analysis supports five subgenera (Fig. 15): *Heraclides*, *Calaides*, *Troilides*, *Priamides*, and Unnamed (consists of *Heraclides hyppason*). Below the subgenus level, we also see instructive species groups, for instance, the Giant Swallowtails: Eastern (*Heraclides cresphontes*), Western (*Heraclides rumiko*), False (*Heraclides homothoas*), and Jamaican (*Heraclides melonius*), form a distinct cluster that can be recognized (Figs 15, 16). While we are not aiming to name every node in the tree, this three-level system (genus, subgenus, species group) for Papilionini covers major phylogenetic clades and may be sufficient.

Third, the most important utility about using the three genera instead of *Papilio* s.l. is the gain of a taxonomic hierarchy level to describe complex phylogenetic relationships within Papilionini. When *Papilio* s.l. is used, it equates to the tribe (tribe = one genus) and we essentially lose a classification level and thus the ability to describe the finer phylogenetic structure of the tree by names. For all these reasons, we think that the simple three-genus system of the native New World Papilionini will stand the test of time.

### Species vs. subspecies

In contrast to genus, species is a more objective biological category. A number of species concepts has been proposed (De Queiroz 2007), with the most popular one being biological. A species is a group of populations capable of interbreeding, but reproductively isolated from all other such groups (Mayr 1963: 12, 14). However, if this concept was truly objective and evolutionary processes stood still, there would be few arguments about species boundaries. Due to ongoing speciation, some groups of populations are in transition. It is very challenging, if not impossible, to decide if they fully crossed the speciation barrier, i.e., developed certain incompatibilities that prevent interbreeding. Additional complexities are caused by the fact that many different species can readily hybridize when opportunities allow (Mallet 2008, Zinner et al. 2011). It is frequently difficult to determine whether the hybrids are characterized by reduced fitness and what amount of fitness loss in hybrids equates complete speciation. Moreover, recent developments in genomics suggest that inter-species hybridization may play a very significant role in evolution, giving an opportunity for closely related species to exchange advantageous genes, e.g., those responsible for mimicry (*Heliconius* Genome Consortium 2012).

While hybridization experiments followed by fitness measurements in hybrids and backcrosses may be decisive in delineating species boundaries, phenotypic differences and genetic divergence are used as more practical criteria. If two populations of the same species have spent significant time in isolation, mutations randomly accumulating in them are likely to cause incompatibilities upon interbreeding, leading to speciation. Some mutations may also cause phenotypic effects, allowing researchers to recognize species by morphological characters. Gene regions rich in neural mutations, such as the COI barcode, are used as yardsticks to estimate divergence between populations. Larger divergence between populations indicates higher chance of speciation. No universal threshold for divergence to mean speciation is possible. Recently formed species may have identical DNA barcodes. High barcode variability within population may lead to conspecific individuals with large barcode differences. To derive sensible conclusions, comparison of barcode variation within and between populations is necessary. Since similar evolutionary mechanisms frequently occur in related organisms, evaluation of barcode variability across the genus is desirable. Finally, correlation between DNA differences and morphological differences is most effective for delineation of species.

In many animals, allopatric populations of the same species characterized by measurable morphological differences, such as those in shapes and colors, are frequently named as subspecies. Typically, subspecies diverged in morphology very recently. Therefore, differences between their DNA barcodes are small compared to those between species. Some of these subspecies are on a path to speciation. Given longer time, and thus more mutations accumulating in the DNA barcode, reproductive incompatibility between these populations will arise. Random extinctions of various populations prune phylogenetic tree and lead to formation of discrete clades that form various clusters. Comparative analysis of these clades and clusters suggests taxonomic hypotheses.

We applied these ideas to selected Neotropical representatives of the tribe Papilionini (Fig. 15–17). We see that differences between subspecies (nodes leading to subspecies are colored green) are mostly within 1.0%. For instance, *Pterourus glaucus glaucus* (Linnaeus, 1758) and *Pterourus glaucus maynardi* (Gauthier, 1984) barcodes differ by 0.2%, or just 1 base pair. On the other hand, differences between species (nodes leading to species are colored red) are typically above 2%. For example, barcode of *Pterourus glaucus glaucus* differs from barcode of *Pterourus canadensis* (Rothschild & Jordan, 1906) by 2.1%, or 14 bp. We noticed several instances of taxa previously treated as subspecies with barcode differences between 2.5% and 5.2% from their respective nominate subspecies (names highlighted orange in Fig. 15). Analysis of their genitalia revealed differences comparable in magnitude to those characteristic of species. Combining the evidence from wing patterns, genitalia and DNA barcodes, we proposed species status for these taxa, as detailed in the Results section.

DNA barcodes of *Heraclides cresphontes* and *Heraclides rumiko* show less than 0.5% difference within each species, but differ by 2.9% between them (Figs 15–17). This difference is comparable to those between other pairs of closely related species in Papilionini, e.g., *Heraclides ornythion* vs. *Heraclides pallas* (2.6%), *Heraclides androgeus* vs. *Heraclides thersites* (2.9%), and *Heraclides multicaudata* vs. *Heraclides canadensis* (2.8%), but significantly larger than differences between subspecies, e.g., of *Heraclides androgeus* (0.3%-1.1%), *Heraclides torquatus* (0.6%-1.1%),and *Heraclides anchisiades* (<0.2%). *Heraclides cresphontes* and *Heraclides rumiko* differ in male genitalia, and most specimens can be told apart by spots vs. stripes on the neck, wing shape, and wing patterns. Therefore we proposed *Heraclides rumiko* as a species. However, it is clear that it is a close sister to *Heraclides cresphontes*, and the two together may be considered a superspecies (Amadon 1966).

Distribution ranges of *Heraclides cresphontes* and *Heraclides rumiko* overlap in central Texas, mostly from Austin to Houston and San Antonio. The two species almost certainly hybridize where they meet. We see that some individuals from the overlap zone show intermediate characteristics and are probable hybrids (Fig. 11E). Despite probable interbreeding, the two taxa maintain integrity and do not absorb each other expanding the overlap zone, which suggests certain reproductive isolation. It is likely that their hybrids are characterized by reduced fitness, preventing the expansion of the overlap zone and free mixing of individuals across the entire ranges of the two species. The absence of broader overlap zone between the two haplotypes and the lack of variability in DNA barcodes within each species over thousands of miles is congruent with the genomic integrity species concept of Sperling (2003). This situation reminds one of the relationships between *Pterourus canadensis* and *Pterourus glaucus*, two species showing a 2.1% difference in the DNA barcode and diverged about 600,000 years ago (Zhang et al. 2013). This pair also has an overlap zone with frequent hybridization between species. Examples of different animal species that hybridize in parts of their ranges are not uncommon, and exist even in vertebrates. For instance, coyotes can interbreed with several species of wolves and descendants of these hybrids are known as coywolves (Hailer and Leonard 2008). Further study of how *Heraclides cresphontes* and *Heraclides rumiko* interact in the overlap zone is expected to yield insights into the mechanisms of speciation, isolation and maintenance of species integrity in the presence of hybridization.

Ultimately, there is no proof, but a hypothesis—or prediction—that we think has a better chance of standing the test of time. Given all the information we assembled, our bet is on the species (and not subspecies) status of *Heraclides rumiko*, which offers a treatment more consistent with how other Papilionini are currently classified (Lamas 2004, Pelham 2008, Warren et al. 2014).

As a summary, we observe three levels of differentiation at and near the species level. First, there are clusters of populations with small genetic differences between them (mostly within 1% in COI barcodes, sometimes no difference at all), but certain geographic differences in wing patters. These populations could be defined as subspecies. Next, there are groups with larger genetic differences (typically above 2% in COI barcodes, but could be less), frequently characterized by measurable differences in genitalia. These groups could be called species. Finally, several mostly allopatric species characterized by closely related phenotypes form very distinct genotypic groups (usually more than 5% in COI barcodes) could be termed a superspecies. All these levels are seen in *Heraclides* (Fig. 15).

### Evolutionary speculations

The 3% difference in DNA barcodes of *Heraclides cresphontes* and *Heraclides rumiko* suggests that the two species diverged between 1 and 3 million years ago (April et al. 2013, Papadopoulou et al. 2009, Zhang et al. 2013). We hypothesize that the speciation of the H. cresphontes group is linked to the Pleistocene glaciation (Fig. 25). Prior to glaciation, certain events lead to formation of the H. thoas group ancestor, probably the southern lineage, and the H. cresphontes group ancestor, probably the northern lineage inhabiting the territory of present-day USA. Formation of ice sheets from the north and colder climate gradually drove *Heraclides cresphontes* populations south, dividing them into at least two groups. One group escaped into Mexico, and the other one was cornered in Florida and the Caribbean Islands, and, though them, went into South America. Due to lower sea levels, the Islands were larger and more accessible from the continent than they are today. Geographic isolation of these *Heraclides cresphontes*-like populations resulted in the four species. Populations on Jamaica lead to *Heraclides melonius*. South American populations speciated into *Heraclides homothoas*. Floridian populations gave rise to *Heraclides cresphontes*. Mexican, and probably the largest, segments developed into *Heraclides rumiko*. With the ice retreat, species started to spread north. *Heraclides homothoas* invaded Central America, *Heraclides cresphontes* took most of the former range of the H. cresphontes group ancestor in eastern US, and met with *Heraclides rumiko* in central Texas.

### Limited dispersal and a need for USDA regulation

In our medium-scale barcoding study we didn’t see any significant invasion of *Heraclides cresphontes* and *Heraclides rumiko* into each other’s ranges (Fig. 18). Eastern populations were *Heraclides cresphontes* and southwestern populations were *Heraclides rumiko*. Specimens with both barcodes were present only in central Texas. However, these butterflies are strong fliers and are known to travel long distances, even being somewhat migratory (Pyle 1981, Scott 1986). Most of the migratory movements seem to be directed north. One may assume that *Heraclides rumiko* might fly from Texas northwards into the Great Plains. Nevertheless we have not found any *Heraclides rumiko* specimens northeast of Texas. The northward movement in California seems to be also rare, perhaps due to the drier climate and lack of the host plants. Equally, we do not observe very frequent invasion of *Heraclides cresphontes* to the west. Apparently, such dispersals are not common, and the two species remain mostly confined to their respective ranges. However, there is some indication that a limited dispersal takes place, shown by the two barcoded records outside the regular range of the two species: northern WY and northeastern CO, which were *Heraclides cresphontes* and *Heraclides rumiko* by the barcodes, respectively.

“Giant Swallowtail” is one of the species used in butterfly release ceremonies across the US. USDA lists *Heraclides cresphontes* as one of the nine species of butterflies that can be transported across state lines and released into the wild under a permit (USDA 2012). Immature stages of *Heraclides cresphontes* are available for purchase on the internet, ready to be shipped from one region of the country to another. Now, since we have shown that *Heraclides cresphontes* is the species that is confined in its distribution to the eastern US (east of 100^th^ Meridian), and *Heraclides rumiko* is the southwestern US species, east-west movements of Giant Swallowtails should be controlled similarly to the Monarch (*Danaus plexippus* (Linnaeus, 1758)). In fact, we think that the Giant Swallowtail regulation will be more useful than the Monarch regulation, because of limited natural dispersal of the two *Heraclides* species. It may be important to discourage transport of Giant Swallowtails across 100^th^ Meridian to prevent unnecessary mixing of the two species. That said, we do not favor the extreme step of shutting releases down, and firmly believe in collaboration between butterfly farmers, environmentalists, enthusiasts, and researchers. Butterfly releases increase awareness of nature and invertebrates, especially among people who may not otherwise be interested in insects. Thus releases serve an educational value, which should not be let go. Unless release practice reaches astronomically large proportions, their impact on natural insect populations will remain negligibly small.

## Supplementary Material

XML Treatment for Heraclides rumiko

## Supplementary materials

Supplementary material 1 Supplementary Table 1 Data type: specimen data. Explanation note: Data for specimens with DNA sequences determined in this study. This dataset is made available under the Open Database License (http://opendatacommons.org/licenses/odbl/1.0/). The Open Database License (ODbL) is a license agreement intended to allow users to freely share, modify, and use this Dataset while maintaining this same freedom for others, provided that the original source and author(s) are credited.Kojiro Shiraiwa, Qian Cong, Nick V. Grishin
